# Deciphering the multifaceted role of *EXO1* in female-related cancers: implications for prognosis and therapeutic responsiveness

**DOI:** 10.3389/fimmu.2025.1591505

**Published:** 2025-05-12

**Authors:** Cong Yu, Guoying Wu

**Affiliations:** School of Life Sciences, Qilu Normal University, Jinan, China

**Keywords:** *EXO1*, female-related cancers, prognostic biomarkers, tumor immune microenvironment, drug sensitivity prediction

## Abstract

**Background:**

Aberrant function or overactivation of exonuclease 1 (*EXO1*) may be associated with cancer tumor development, drug resistance, and response to immunotherapy in female-related cancers.

**Methods:**

By analyzing RNA-sequencing data from The Cancer Genome Atlas database, combined with validation through quantitative polymerase chain reaction experiments, we explored the expression levels of *EXO1* in breast cancer (BRCA) cell lines and assessed its multidimensional roles in various female-related cancers.

**Results:**

Our experiments revealed elevated expression of *EXO1* in BRCA cell lines, consistent with the RNA-sequencing data. The high expression of *EXO1* is associated with poor prognosis in various female-related cancers, especially in BRCA and UCEC. It significantly correlates with clinical and pathological characteristics. In specific cancer subtypes like the basal-like subtype of BRCA, high *EXO1* expression is associated with a better prognosis. Genetic mutation analysis indicates a higher frequency of *EXO1* gene mutations in uterine sarcoma and BRCA. DNA methylation levels may play a role in the regulation of *EXO1* gene expression in some cancers. *EXO1* expression is correlated with various factors within the tumor immune microenvironment and may be associated with the sensitivity to anticancer drugs.

**Conclusion:**

*EXO1* exhibits multidimensional roles in female-related cancers as a prognostic biomarker and potentially influences tumor immune therapy responses and drug sensitivities. Further studies are needed to fully understand the complex mechanisms underlying these associations and to explore potential therapeutic strategies targeting *EXO1*.

## 1 Introduction

In recent years, there has been a continuous increase in the number of patients with cancer globally. By 2020, the number of patients with cancer worldwide had reached 19.3 million, with 9.228 million being women, representing 47.8% of the total patient population (1). The most prevalent types of cancer in women are breast cancer (BRCA), cervical cancer (CESC), uterine corpus endometrial cancer (UCEC), ovarian cancer (OV), thyroid cancer (THCA), uterine sarcoma (UCS), etc. These cancers account for 44.5% of all female-related cancer cases and have a mortality rate of 31.3% (1). BRCA ranks among the most prevalent malignancies in women. In 2024, it was projected that there would be 310,720 new cases of female BRCA, surpassing lung cancer 118,270) and becoming the leading type of cancer in the United States, representing approximately 32% of all new cancer cases (2). In China, there are 269,000 new cases of BRCA and 70,000 deaths annually, placing it at the top with regard to incidence and fourth in terms of mortality among female-related malignancies (3). CESC (including squamous cell carcinoma and adenocarcinoma), UCEC, and OV are prevalent gynecologic malignancies. The incidence of these cancers has been on the rise over time, with a gradual decrease in the average age at which they occur (4–6). The incidence of THCA in females has risen rapidly since 2000, and with an incidence 3-4 times higher in females compared to males (7–9). Therefore, similar to breast cancer, THCA is regarded as a female-related cancer in our study.

Exonuclease 1 (*EXO1*), a 5′-3′ exonuclease, exists in two isoforms (i.e., *EXO1a* and *EXO1b*), which share similar functions (10). The EXO1 protein exhibits both 5’ end-3’ end exonuclease activity and 5’ end structure-specific endonuclease activity. It is classified within the radiation-sensitive 2/xeroderma pigmentosum complementation group G (*Rad2/XPG*) family (11). *EXO1* plays a vital role in various DNA repair processes, such as mismatch repair, translation synthesis, nucleotide excision repair, DNA double-strand break repair, meiotic recombination repair, and telomere maintenance (12). Research has demonstrated that the absence of the MutL homolog 1 (*MLH1*) subunit of MutLα disrupts its specific regulation of EXO1 during DNA repair, resulting in uncontrolled DNA excision, which encompasses elevated single-stranded DNA formation, depletion of replication protein A (RPA), DNA breaks, and abnormal DNA repair intermediates (13). While DNA processing is crucial for repair, excessive activity can cause heightened genome instability and disruptions to cellular function (14). Considering that platinum-based chemotherapeutic drugs primarily induce DNA damage, the capacity of tumor cells to repair such damage plays a pivotal role in their resistance to these drugs. Cancer recurrence and chemotherapy resistance are frequent factors contributing to mortality among patients with cancer. Research has revealed that deficiency of the *EXO1* gene can heighten susceptibility and chemotherapy resistance in various malignancies, including BRCA, OV, and lung cancer. Additionally, its polymorphism can serve as a prognostic indicator for squamous cell carcinoma, epithelial OV, nonsmall cell lung cancer, pancreatic cancer, and head and neck cancer (15, 16). Disruption of the *EXO1* gene resulted in heightened microsatellite instability in cells and increased tumor susceptibility in mice (17). Hence, the EXO1 protein likely plays a significant role in cancer chemoresistance and holds promise as a therapeutic target for enhancing chemosensitivity in drug-resistant patients. Nevertheless, further investigation is required to elucidate the precise signaling pathway through which the EXO1 protein operates in DNA damage repair mechanisms and its involvement in tumor chemoresistance.

This study comprehensively examined the expression profile, genetic alterations, and molecular functions of *EXO1*, along with its correlation with clinicopathological characteristics, prognostic significance, and infiltration of cancer-associated immune cells in various types of female-related tumors.

## 2 Materials and methods

### 2.1 Cell culture

MCF-7 cells were cultured in cell-specific culture medium (CM-0149, Procell Co., Ltd, Wuhan, China). MCF-10A cells were cultured in specific epithelial culture medium (CM-0525, Procell Co., Ltd). All cells were maintained at 37°C in a humidified atmosphere composed of 95% air and 5% CO_2_; the media were changed every 2 days.

### 2.2 Real-time quantitative polymerase chain reaction

RNA was extracted from the cells using AG RNAex Pro reagent (AG21101, Accurate Biotechnology Co., Ltd, Changsha, China) and reversely transcribed to cDNA using an RT Kit (AG11728, Accurate Biotechnology Co., Ltd). Subsequently, the mRNA levels of the targeted gene (*EXO1*) relative to those of control glyceraldehyde-3-phosphate dehydrogenase (*GAPDH*) were quantified by qPCR using specific primers synthesized by Sangon Biotech (Shanghai, China). The sequences of the primers were *EXO1*-1: forward: 5’-CTGAAGTGTTTGTGCCTGAC-3’ and reverse: 5′-GTGGGTGGTGAAATGGTC-3’; *EXO1*-2: forward: 5’- TACTGTGGGAGTGGAACG-3’ and reverse: 5′- TCCATTTACCAGGTCAGG-3’; *EXO1*-3: forward: 5’- ATTGCCTCGTGGCTCCCTAT-3’ and reverse: 5′- ACCCGTTGATGTAATCCTCTGG-3’.

### 2.3 Data acquisition and gene expression analyses

RNA-sequencing (RNA-seq) data along with patient-specific clinical information, were obtained from The Cancer Genome Atlas (TCGA) database (https://prtal.gdc.cancer.gov/) for patients with cancer enrolled in the BRCA, CESC, OV, THCA, UCEC, and UCS projects. RNA-seq data were processed uniformly using TCGA Toil application (18). Gene Expression Profiling Interactive Analysis (GEPIA; gepia2.cancer-pku.cn) was employed to conduct expression analysis by generating box plots comparing the expression levels of *EXO1* in cancer samples and healthy control samples (19). Wilcoxon rank sum and signed rank tests were utilized to compare the expression levels of *EXO1* between tumor samples and paired or unpaired control samples. To assess the discriminatory power of *EXO1* expression levels (high or low) in distinguishing tumor samples from control samples, receiver operating characteristic (ROC) analysis was conducted using the “pROC” package (20). This study was carried out following the publication guidelines established by TCGA.

### 2.4 Clinicopathological characteristics analyses

Differences in clinicopathological characteristics were assessed between the high and low *EXO1* expression groups using various statistical tests, such as Pearson’s χ2 test, Fisher’s exact test, or Wilcoxon rank sum test. Logistic regression analysis was applied to evaluate the association between *EXO1* expression levels and clinicopathological variables among female patients with cancer. Visualization of the statistical data was accomplished using the R package “ggplot2”.

### 2.5 Survival and prognostic analyses

The objective was to evaluate the potential of *EXO1* as a predictor of overall survival (OS), disease-specific survival (DSS), and progression-free interval (PFI) in female patients with cancer and subgroups. Survival differences between the high and low *EXO1* expression groups were assessed, and Kaplan–Meier curves were constructed using data from TCGA. The “survival” package and the “survminer” package in R were utilized for these analyses. Univariate and multivariate Cox regression analyses were performed to identify independent prognostic factors associated with survival. Variables with *P*-values <0.1 in the univariate analysis were included in the multivariate Cox regression model. *Hazard ratios* (HR) with their corresponding 95% confidence intervals (CI) were calculated to estimate the risk associated with each factor.

### 2.6 Nomogram construction and evaluation

Following a previous Cox multifactorial regression model, nomograms were constructed using the “rms” package in R to identify independent prognostic factors. Calibration plots were subsequently constructed to assess the predictive accuracy of the nomogram according to the concordance between predicted and actual OS, DSS, and PFI at 1, 3, and 5 years. A concordance index was calculated using a bootstrap approach with 1,000 resamples to determine the discriminatory power of the nomogram (21). A time-dependent survival ROC curve was produced using the “timeROC” and “ggplot2” packages in R to assess the predictive value of *EXO1* expression for 1-, 3-, and 5-year survival in female-related cancers in TCGA (22).

### 2.7 Genetic alteration analyses

The genetic alterations of *EXO1* gene were analyzed using the cBioPortal (www.cbioportal.org/) and TIMER2.0 (http://timer.cistrome.org/) in databases of female-related cancers. Kaplan–Meier curves were drawn based on the log-rank test using cBioPortal to analyze the different prognostic significance between *EXO1* altered and unaltered groups among various female-related cancers (23).

### 2.8 DNA methylation status analyses

The UALCAN online tool (http://ualcan.path.uab.edu/) was employed to analyze and compare the methylation levels of *EXO1* in female-related cancers and normal tissues using TCGA data (24). The association between different DNA methylation probes and *EXO1* expression was assessed using the “ggplot2” package in R. Additionally, the beta value, *P*-value, and Pearson correlation coefficient were evaluated for each individual probe. The prognostic value of the *EXO1* methylation level in female-related cancers was analyzed using the SurvivalMeth online tool (http://bio-bigdata.hrbmn.edu.cn/survivalmeth/) with TCGA data (25).

### 2.9 Differentially expressed genes and enrichment analysis

Tumor samples were classified into high and low expression subgroups based on the median *EXO1* expression. DEGs were identified from HTSeq-Counts using the DESeq2 software, with thresholds of |log2 fold change| >2 and adjusted *P*-values <0.01 (26). Results of DEG analysis are presented as volcano plots. The R package ClusterProfiler (3.14.3) was utilized for Gene Ontology (GO) classification, including biological process, cellular components, and molecular function, as well as for Kyoto Encyclopedia of Genes and Genomes (KEGG) pathway enrichment analysis of DEGs between the high and low *EXO1* expression groups (27). Terms with *P*-values <0.05 after adjustment using the Benjamini and Hochberg method were considered significant. The R package ClusterProfiles (3.14.3) was used to perform gene set enrichment analysis (GSEA) between high and low *EXO1* expression groups. The expression level of *EXO1* was used as a phenotype label, and enriched pathways were identified according to |normalized enrichment score| >1, adjusted *P*-values <0.05, and false discovery rate q-value <0.25. Pearson correlation analysis was performed between *EXO1* and all other molecules, and the top 100 most significantly correlated genes were selected. The overlapping genes across all groups were identified, and the intersection results were visualized as a flower plot using the ggplot2 package (v3.4.2). Protein-protein interaction (PPI) networks were subsequently analyzed through the STRING database (https://string-db.org, v12.0) with high-confidence interaction thresholds (score ≥0.7).

### 2.10 Cancer immune analysis

The TISIDB database was used to analyze the correlations between *EXO1* expression and immune subtypes in female-related cancers (28). Immune infiltration analysis of 24 distinct immune cell types within tumor samples was performed using the single-sample GSEA method with gene set variation analysis software in the R environment. Relative enrichment scores were calculated for each tumor sample based on the characteristic genes of these 24 immune cell types (29). Spearman correlation analysis was employed to assess associations between *EXO1* expression and the infiltration of each immune cell type. The Wilcoxon rank sum test was used to compare cell immune infiltration between high and low *EXO1* expression groups. The potential association of *EXO1* expression with immune inhibitors, immune stimulators, chemokines, and chemokine receptors in female-related cancers were determined using Spearman’s rank correlation test.

### 2.11 Drug sensitivity analysis

The relationship between the sensitivity of hundreds of drugs and the relative expression levels of *EXO1* was analyzed using The Genomics of Drug Sensitivity of Cancer (GDSC) and The Cancer Therapeutics Response Portal (CTRP) database (http://bioinfo.life.hust.edu.cn/GSCA/#/).

## 3 Results

### 3.1 Expression of *EXO1* in multiple female-related cancers

**Figure 1:**
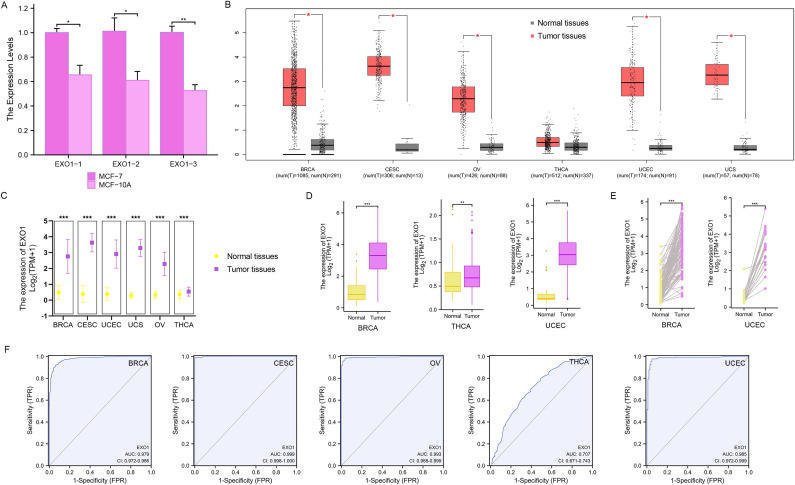
*EXO1* mRNA expression levels in female-related cancers. **(A)** QPCR analysis of the relative levels of *EXO1* mRNA transcripts in MCF-7 cells and MCF-10A cells. **(B)** Analysis of *EXO1* expression in female-related tumor tissues and normal tissues using GEPIA. **(C)** *EXO1* mRNA expression in tumor samples and normal samples from combined TCGA and GTEx datasets. **(D)** *EXO1* mRNA expression in tumor samples compared to adjacent normal samples from TCGA. **(E)** Differences in *EXO1* mRNA expression between individual tumor samples and paired adjacent normal tissue samples from TCGA. (*P < 0.05; **P < 0.01; ***P < 0.001). **(F)** ROC curves assessing the efficiency of *EXO1* expression for distinguishing tumor tissues (BRCA, CESC, OV, THCA, and UCEC) from non-tumor tissues using the combined TCGA and GTEx dataset. BRCA, breast cancer; CESC, cervical cancer; *EXO1*, exonuclease 1; GEPIA, Gene Expression Profiling Interactive Analysis; GTEx, Genotype-Tissue Expression Project; OV, ovarian cancer; QPCR, quantitative polymerase chain reaction; ROC, receiver operating characteristic; TCGA, The Cancer Genome Atlas; THCA, thyroid cancer; UCEC, uterine corpus endometrial cancer.

The qPCR analyses revealed that the relative levels of *EXO1* expression in MCF-7 cells were significantly higher than those in MCF-10A cells (*P* < 0.001) (**Figure 1A**). The expression of *EXO1* in multiple female-related cancers, including BRCA, CESC, OV, THCA, UCEC, and UCS, was analyzed using GEPIA. The results demonstrated that *EXO1* expression levels were significantly higher in patients with BRCA, CESC, OV, UCEC, and UCS, compared to healthy controls (*P* < 0.001) (**Figure 1B**). The expression of *EXO1* in these female-related cancers was further analyzed using R based on data from TCGA and other databases. The analysis revealed that *EXO1* was significantly upregulated in tumor tissues versus normal tissues for BRCA, CESC, OV, THCA, UCEC, and UCS based on a combined dataset from the Genotype-Tissue Expression Project (GTEx) and TCGA (*P* < 0.001) (**Figure 1C**). Moreover, *EXO1* mRNA expression was found to be elevated in tumor tissues compared to paracancerous tissues specifically in BRCA, THCA, and UCEC using data from TCGA database (*P* < 0.001) (**Figure 1D**). Furthermore, when comparing individual tissue samples to their corresponding adjacent tissues within the BRCA and UCEC datasets from TCGA, elevated *EXO1* mRNA expression was observed (*P* < 0.001) (**Figure 1E**). To evaluate the discriminatory capability of *EXO1* expression levels between tumor and non-tumor tissues, ROC analysis was performed using combined TCGA and GTEx data. The areas under the curve (AUC) were 0.979 for BRCA, 0.999 for CESC, 0.993 for OV, 0.707 for THCA, and 0.985 for UCEC (**Figure 1F**). These results suggest that *EXO1* is significantly upregulated in a variety of tumors related to females and possesses a good ability to distinguish between tumor and non-tumor tissues.

### 3.2 Associations of *EXO1* expression levels with clinicopathologic characteristics

Given the high expression of *EXO1* in female-related cancers, we investigated the associations between *EXO1* expression and clinicopathological characteristics of patients based on TCGA data. **Table 1** presents the significant differences in various clinicopathological characteristics between patients with high or low *EXO1* expression levels in female-related cancers. In patients with BRCA, there were significant differences in T stage (*P* < 0.001), pathologic stage (*P* = 0.008), race (*P* < 0.001), age (*P* < 0.001), histologic type (*P* < 0.001), progesterone receptor (PR) status (*P* < 0.001), estrogen receptor (ER) status (*P* < 0.001), human epidermal growth factor receptor 2 (HER2) status (*P* < 0.001), PAM50 (*P* < 0.001), anatomic neoplasm subdivisions (*P* = 0.001), OS event (*P* = 0.046), and DSS event (*P* = 0.034) between different *EXO1* expression groups. In patients with UCEC, significant differences were observed in weight (*P* = 0.004), histological type (*P* < 0.001), histologic grade (*P* < 0.001), OS event (*P* = 0.017), and PFI event (*P* = 0.003) between different *EXO1* expression groups. THCA patients with different *EXO1* expression levels displayed significant differences in histological type (*P* = 0.001) and PFI event (*P* = 0.031). However, no significant differences were found between the high and low *EXO1* expression groups in CESC, OV, and UCS.

**Table 1:** Clinicopathological characteristics of female related cancers patients with high- and low- EXO1 expression.

Cancer type	Characteristic	EXO1 expression		*P*	Statistic Value
		Low	High		
BRCA	n	541	542		
	T stage, n (%)			< 0.001	29.81^a^
	T1	174 (16.1%)	103 (9.5%)		
	T2	280 (25.9%)	349 (32.3%)		
	T3	74 (6.9%)	65 (6%)		
	T4	12 (1.1%)	23 (2.1%)		
	Pathologic stage, n (%)			0.008	11.81 ^a^
	Stage I	111 (10.5%)	70 (6.6%)		
	Stage II	290 (27.4%)	329 (31%)		
	Stage III	119 (11.2%)	123 (11.6%)		
	Stage IV	9 (0.8%)	9 (0.8%)		
	Race, n (%)			< 0.001	26.62 ^a^
	Asian	16 (1.6%)	44 (4.4%)		
	Black or African American	75 (7.5%)	106 (10.7%)		
	White	417 (42%)	336 (33.8%)		
	Age, n (%)			< 0.001	11.49 ^a^
	<=60	272 (25.1%)	329 (30.4%)		
	>60	269 (24.8%)	213 (19.7%)		
	Histological type, n (%)			< 0.001	66.24 ^a^
	Infiltrating Ductal Carcinoma	327 (33.5%)	445 (45.5%)		
	Infiltrating Lobular Carcinoma	153 (15.7%)	52 (5.3%)		
	PR status, n (%)			< 0.001	
	Negative	100 (9.7%)	242 (23.4%)		
	Indeterminate	2 (0.2%)	2 (0.2%)		
	Positive	415 (40.1%)	273 (26.4%)		
	ER status, n (%)			< 0.001	
	Negative	46 (4.4%)	194 (18.7%)		
	Indeterminate	0 (0%)	2 (0.2%)		
	Positive	471 (45.5%)	322 (31.1%)		
	HER2 status, n (%)			< 0.001	14.33 ^a^
	Negative	293 (40.3%)	265 (36.5%)		
	Indeterminate	7 (1%)	5 (0.7%)		
	Positive	56 (7.7%)	101 (13.9%)		
	PAM50, n (%)			< 0.001	428.14 ^a^
	Normal	35 (3.2%)	5 (0.5%)		
	LumA	434 (40.1%)	128 (11.8%)		
	LumB	37 (3.4%)	167 (15.4%)		
	Her2	15 (1.4%)	67 (6.2%)		
	Basal	20 (1.8%)	175 (16.2%)		
	Anatomic neoplasm subdivisions, n (%)			0.001	10.58 ^a^
	Left	254 (23.5%)	309 (28.5%)		
	Right	287 (26.5%)	233 (21.5%)		
	OS event, n (%)			0.046	4 ^a^
	Alive	477 (44%)	454 (41.9%)		
	Dead	64 (5.9%)	88 (8.1%)		
	DSS event, n (%)			0.034	4.48 ^a^
	Alive	503 (47.3%)	475 (44.7%)		
	Dead	33 (3.1%)	52 (4.9%)		
UCEC	n	276	276		
	Weight, n (%)			0.004	8.26 ^a^
	<=80	105 (19.9%)	138 (26.1%)		
	>80	160 (30.3%)	125 (23.7%)		
	Histological type, n (%)			< 0.001	15.55 ^a^
	Endometrioid	225 (40.8%)	185 (33.5%)		
	Mixed	10 (1.8%)	14 (2.5%)		
	Serous	41 (7.4%)	77 (13.9%)		
	Histologic grade, n (%)			< 0.001	58.43 ^a^
	G1	78 (14.4%)	20 (3.7%)		
	G2	72 (13.3%)	48 (8.9%)		
	G3	122 (22.6%)	201 (37.2%)		
	OS event, n (%)			0.017	5.65 ^a^
	Alive	240 (43.5%)	218 (39.5%)		
	Dead	36 (6.5%)	58 (10.5%)		
	PFI event, n (%)			0.003	9.1 ^a^
	Alive	227 (41.1%)	196 (35.5%)		
	Dead	49 (8.9%)	80 (14.5%)		
THCA	n	255	255		
	Histological type, n (%)			0.001	
	Classical	170 (33.3%)	194 (38%)		
	Follicular	67 (13.1%)	34 (6.7%)		
	Other	5 (1%)	4 (0.8%)		
	Tall Cell	13 (2.5%)	23 (4.5%)		
	PFI event, n (%)			0.031	4.66 ^a^
	Alive	236 (46.3%)	220 (43.1%)		
	Dead	19 (3.7%)	35 (6.9%)		

a Chisq.test; others, Fisher.test.

**Figure 2:**
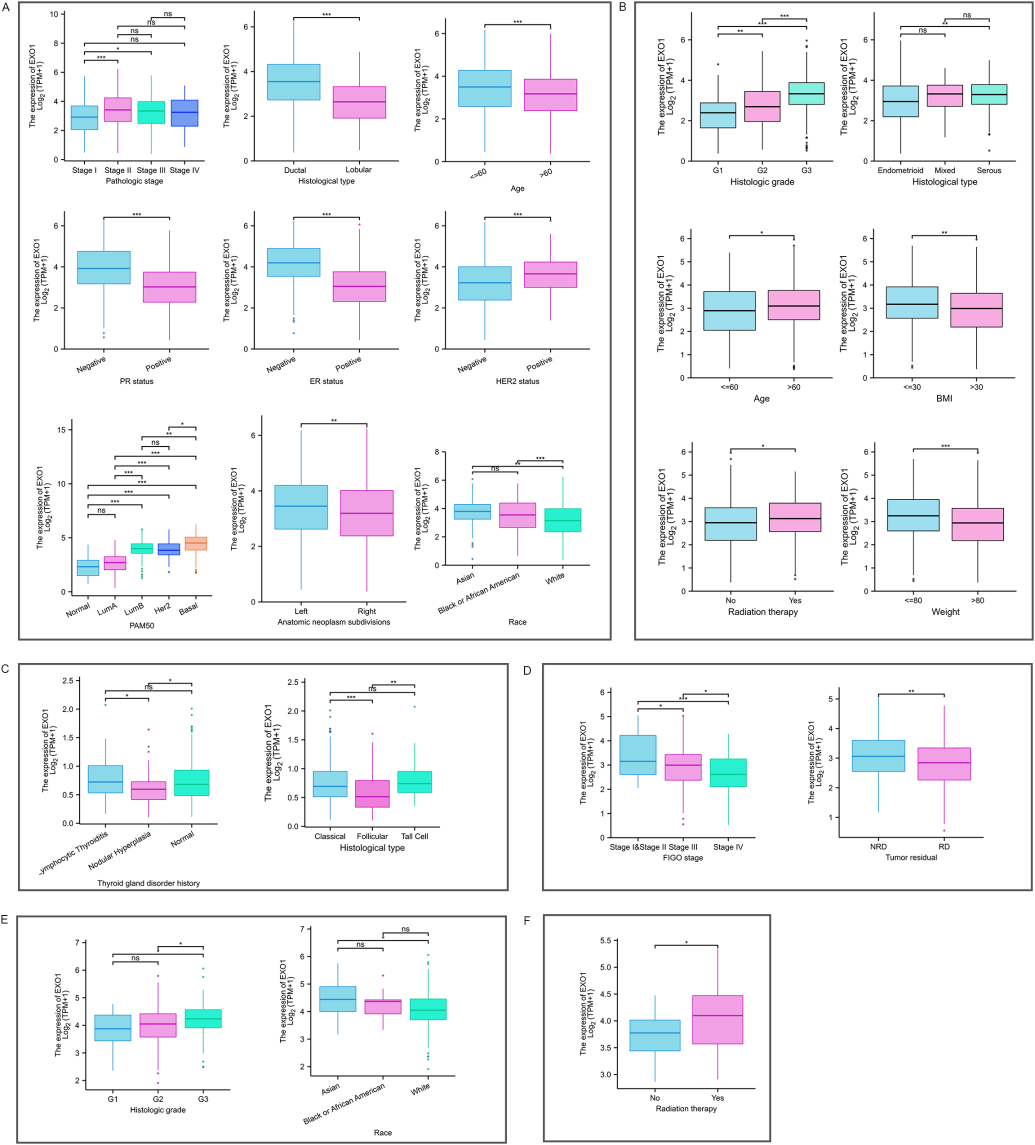
Relationship between *EXO1* expression and various clinicopathological factors. *EXO1* expression in different clinicopathological features in patients with BRCA **(A)**, UCEC **(B)**, THCA **(C)**, OV **(D)**, CESC **(E)**, and UCS **(F)**. (ns: *P* ≥ 0.05; *P < 0.05; **P < 0.01; ***P < 0.001). BRCA, breast cancer; CESC, cervical cancer; *EXO1*, exonuclease 1; ns, no significance; OV, ovarian cancer; THCA, thyroid cancer; UCEC, uterine corpus endometrial cancer; UCS, uterine sarcoma.

The Kruskal–Wallis rank sum test indicated that the mRNA expression levels of *EXO1* were related to pathologic stage, histological type, age, race, PR status, ER status, HER2 status, and PAM50 in patients with BRCA (**Figure 2A**). Expression of *EXO1* was higher in BRCA patients with pathologic stage II/III, infiltrating ductal carcinoma, age ≤60 years, PR-negative, ER-negative, HER2-positive, basal-like of PAM50 classification, and non-White race. In patients with UCEC (**Figure 2B**), higher *EXO1* expression was correlated with higher histologic grade, serous tumors, age >60 years, body mass index >30 kg/m^2^, weight <80 kg, and undergoing radiation therapy. In patients with THCA (**Figure 2C**), the expression levels of *EXO1* were higher in those with a history of lymphocytic thyroiditis in the thyroid gland disorder than in patients with nodular hyperplasia. Additionally, the *EXO1* expression levels were higher in THCA patients with the classical type and tall cell variant compared to the follicular variant. In patients with OV (**Figure 2D**), higher *EXO1* expression showed an inverse correlation with both International Federation of Gynecology and Obstetrics (FIGO) stage and the presence of residual tumor. In patients with CESC (**Figure 2E**), higher *EXO1* expression was associated with higher histologic grade and Asian race. Moreover, in patients with UCS (**Figure 2F**), higher *EXO1* expression was related to radiation therapy.

Logistic regression analysis revealed that *EXO1* expression was significantly associated with T stage, race, age, histological type, PR status, ER status, HER2 status, PAM50 classification, and anatomic neoplasm subdivisions in BRCA. In UCEC, *EXO1* expression was associated with clinical stage, weight, histological type, and histologic grade. In THCA, *EXO1* expression was associated with age, histological type, and a history of thyroid gland disorder. In CESC, *EXO1* expression was associated with histologic grade (**Table 2**). However, *EXO1* expression showed no correlation with clinicopathological characteristics in OV and UCS based on logistic regression analysis.

**Table 2:** Relationship between EXO1 expression levels and clinicopathological characteristics according to logistic regression analyses in female related cancers.

Cancer type	Characteristics	Total (N)	Odds Ratio (OR)	*P*-value
BRCA	T stage (T2&T3&T4 vs. T1)	1080	2.017 (1.526-2.676)	<0.001
	Race (White vs. Asian&Black or African American)	994	0.489 (0.362-0.657)	<0.001
	Age (>60 vs. <=60)	1083	0.655 (0.514-0.833)	<0.001
	Histological type (Infiltrating Lobular Carcinoma vs. Infiltrating Ductal Carcinoma)	977	0.250 (0.175-0.351)	<0.001
	PR status (Positive vs. Negative)	1030	0.272 (0.205-0.358)	<0.001
	ER status (Positive vs. Negative)	1033	0.162 (0.113-0.228)	<0.001
	HER2 status (Positive vs. Negative)	715	1.994 (1.387-2.890)	<0.001
	PAM50 (Her2&Basal vs. LumA&LumB)	1043	11.039 (7.621-16.432)	<0.001
	Anatomic neoplasm subdivisions (Right vs. Left)	1083	0.667 (0.525-0.848)	<0.001
UCEC	Clinical stage (Stage II&Stage III&Stage IV vs. Stage I)	552	1.494 (1.058-2.114)	0.023
	Weight (>80 vs. <=80)	528	0.594 (0.420-0.839)	0.003
	Histological type (Serous vs. Endometrioid)	528	2.284 (1.499-3.521)	<0.001
	Histologic grade (G3 vs. G1&G2)	541	3.634 (2.534-5.254)	<0.001
THCA	Age (>45 vs. <=45)	510	0.696 (0.490-0.986)	0.042
	Histological type (Follicular vs. Classical)	465	0.445 (0.278-0.701)	<0.001
	Thyroid gland disorder history (Nodular Hyperplasia vs. Lymphocytic Thyroiditis)	142	0.439 (0.221-0.858)	0.017
CESC	Histologic grade (G3&G4 vs. G1&G2)	274	1.698 (1.051-2.757)	0.031

a Categorical dependent variable, greater or less than the median expression level.

### 3.3 Prognostic value of *EXO1* expression in UCEC

**Figure 3:**
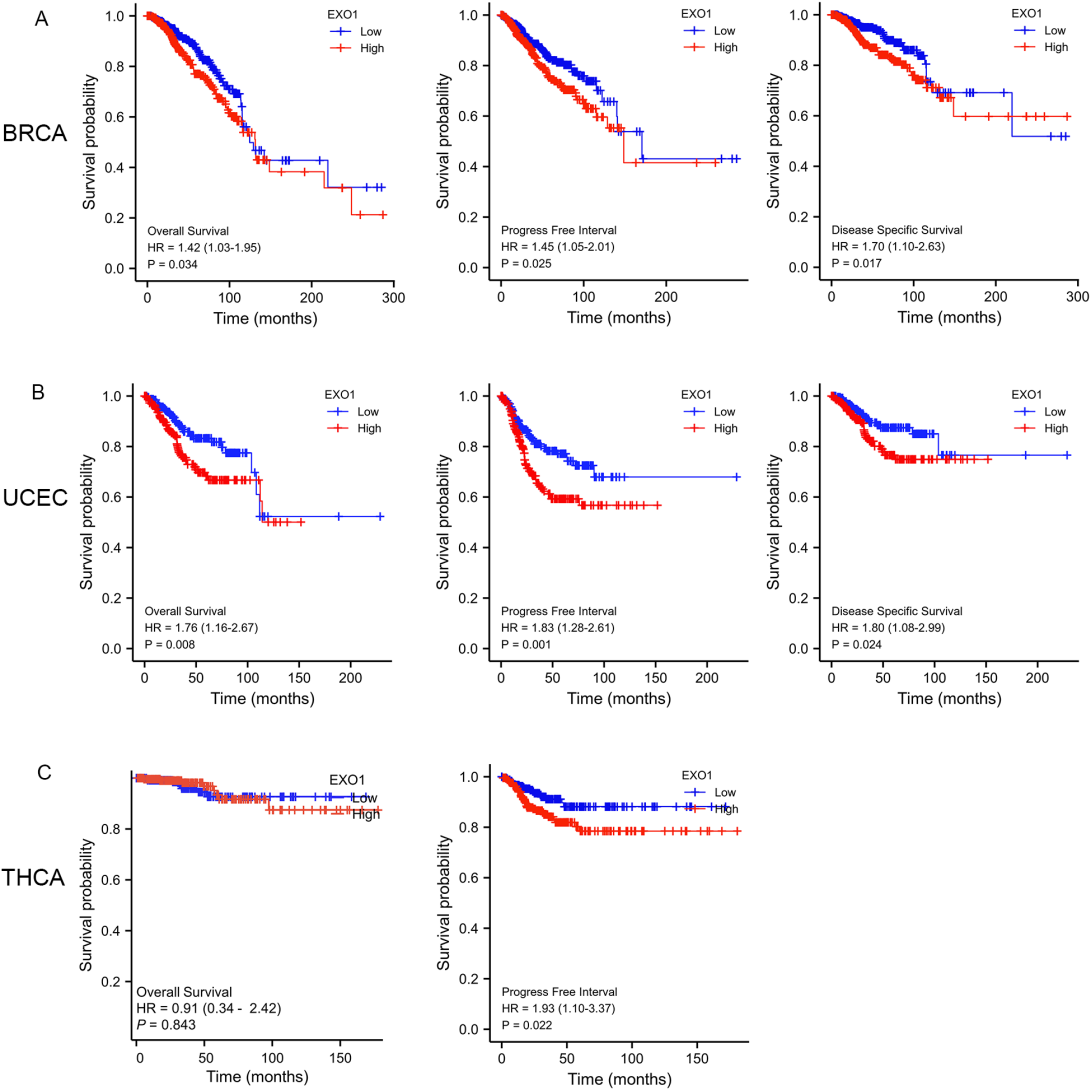
Prognostic prediction values of *EXO1* in female-related cancers. **(A)** Kaplan–Meier analysis indicated poorer OS, PFI, and DSS among BRCA patients with high *EXO1* mRNA expression. **(B)** Kaplan–Meier analysis indicated poorer OS, PFI, and DSS among UCEC patients with high *EXO1* mRNA expression. **(C)** Kaplan–Meier analysis indicated poorer PFI among THCA patients with high *EXO1* mRNA expression. BRCA, breast cancer; DSS, disease-specific survival; *EXO1*, exonuclease 1; HR, *hazard ratio*; OS, overall survival; PFI, progression-free interval; THCA, thyroid cancer; UCEC, uterine corpus endometrial cancer.

To determine the prognostic significance of *EXO1* expression in female-related cancers, we investigated the correlation between *EXO1* expression and patient prognosis. Patients were categorized into high and low *EXO1* expression groups based on their *EXO1* expression levels. Kaplan–Meier analyses revealed that higher *EXO1* expression in patients with BRCA was associated with poorer OS (HR = 1.42 [95% CI: 1.03–1.95], *P* = 0.034), PFI (HR = 1.45 [95% CI: 1.05–2.01], *P* = 0.025), and DSS (HR = 1.70 [95% CI: 1.10–2.63], *P* = 0.017) (**Figure 3A**). Similarly, in patients with UCEC (**Figure 3B**), higher *EXO1* expression was correlated with poorer OS (HR = 1.76 [95% CI: 1.16–2.67], *P* = 0.008), PFI (HR = 1.83 [95% CI: 1.28–2.61], *P* = 0.001), and DSS (HR = 1.80 [95% CI: 1.08–2.99], *P* = 0.024), as well as with poorer PFI (HR = 1.93 [95% CI: 1.10–3.37], *P* = 0.022) in patients with THCA (**Figure 3C**). However, no significant associations were found between *EXO1* expression and prognosis in patients with CESC, OV, and UCS based on data from TCGA database.

Subgroup analysis further revealed that high *EXO1* expression was associated with poor OS in specific subgroups of patients with BRCA, including those with T2 stage, N1 stage, M0 stage, pathologic stage II, luminal B subtype of PAM50 classification, age <60 years, and infiltrating ductal carcinoma histological type (**Supplementary Figures S1A–G**). Additionally, high *EXO1* expression levels were associated with poor PFI in female patients with THCA, as well as in THCA patients with left lobe of neoplasm location, no extrathyroidal extension, residual tumor classification of R0, and multifocal of primary neoplasm focus type (**Supplementary Figures S1H–L**). However, for BRCA patients with basal type of PAM50 classification (**Supplementary Figure S1M**), CESC patients with T2 stage, squamous cell carcinoma of histological type (**Supplementary Figures S1N, O**), and OV patients with FIGO stage IV (**Supplementary Figure S1P**), high *EXO1* expression was associated with better OS.

In addition, high *EXO1* expression was associated with worse OS in UCEC patients with clinical stage I, tumor invasion >50%, open surgical approach, residual tumor classification of R0, and histological type of endometroid (**Supplementary Figures S2A–E**). Furthermore, high expression levels of *EXO1* were correlated with poor OS in patients with UCEC without radiation or hormonal therapy, or without diabetes (**Supplementary Figures S2F–H**). Within specific subgroups, high *EXO1* expression was associated with poorer OS for White patients, or patients aged >60 years, post-menopausal, with height >160 cm, or weighing <80 kg (**Supplementary Figures S2I–M**).

To further illustrate the relationship between *EXO1* expression, clinicopathological parameters, and prognosis in female-related cancers, univariate and multivariate Cox analyses were performed using TCGA data. The results revealed several independent risk factors for OS, PFI, and DSS in different cancer types. For predicting OS, M stage, age, menopause status, and radiation therapy were independent risk factors in BRCA. In UCEC, clinical stage and radiation therapy were independent risk factors. Pathologic stage and residual tumor were independent risk factors in THCA. Primary therapy outcome and tumor status were independent risk factors in OV. In CESC, T stage, N stage, tumor protein p53 (*TP53*) status, and primary therapy outcome were independent risk factors.

Lastly, primary therapy outcome was an independent risk factor in UCS (**Table 3**). Regarding PFI, M stage and PR status were independent risk factors in BRCA. In UCEC, clinical stage, primary therapy outcome, histological type, residual tumor and *EXO1* were identified as independent risk factors. Similarly, M stage and *EXO1* were independent risk factors in THCA. Primary therapy outcome and tumor status were independent risk factors in OV. For CESC, primary therapy outcome was an independent risk factor. *TP53* status and radiation therapy were independent risk factors in UCS (**Supplementary Table S1**). For predicting DSS, N stage, M stage and *EXO1* were identified as independent risk factors in BRCA. In UCEC, clinical stage, primary therapy outcome, histological type, residual tumor and radiation therapy were independent risk factors. Primary therapy outcome and tumor residual were independent risk factors in OV (**Supplementary Table S2**). Therefore, high *EXO1* expression serves as a strong independent predictor of PFI in UCEC and THCA, as well as DSS in BRCA.

**Table 3:** Associations of overall survival (OS) with clinicopathologic characteristics in TCGA patients by univariate and multivariate analyses.

Characteristics	Univariate analysis		Multivariate analysis	
	HR (95% CI)	*P*-value	HR (95% CI)	*P-*value
BRCA				
T stage (T1 vs. T3&T4)	1.953 (1.221-3.123)	0.005	1.310 (0.372-4.606)	0.674
N stage				
N0 vs. N1	1.956 (1.329-2.879)	<0.001	0.740 (0.255-2.150)	0.580
N0 vs. N2	2.519 (1.482-4.281)	<0.001	1.377 (0.251-7.543)	0.712
N0 vs. N3	4.188 (2.316-7.574)	<0.001	3.070 (0.534-17.666)	0.209
M stage (M0 vs.M1)	4.254 (2.468-7.334)	<0.001	18.655 (1.571-221.537)	0.020
Patdologic stage				
Stage I vs. Stage II	1.697 (0.985-2.922)	0.057	3.359 (0.709-15.914)	0.127
Stage I vs. Stage III	2.962 (1.664-5.273)	<0.001	7.399 (0.773-70.798)	0.082
Stage I vs. Stage IV	11.607 (5.569-24.190)	<0.001		
Age (<=60 vs. >60)	2.020 (1.465-2.784)	<0.001	2.582 (1.232-5.411)	0.012
Menopause status (Pre vs. Post)	2.162 (1.300-3.595)	0.003	3.197 (1.089-9.386)	0.034
Radiation tderapy (No vs. Yes)	0.576 (0.394-0.841)	0.004	0.475 (0.236-0.956)	0.037
EXO1 (Low vs. High)	1.416 (1.026-1.955)	0.034	1.583 (0.688-3.641)	0.280
UCEC				
Clinical stage				
Stage I vs. Stage III	3.078 (1.907-4.968)	<0.001	2.689 (1.123-6.442)	0.026
Stage I vs. Stage IV	8.065 (4.488-14.495)	<0.001	2.686 (0.750-9.620)	0.129
Primary tderapy outcome (PD vs. CR)	0.111 (0.060-0.205)	<0.001	0.353 (0.106-1.176)	0.090
Histological type				
Endometrioid vs. Mixed	2.421 (1.036-5.655)	0.041	3.382 (0.899-12.715)	0.071
Endometrioid vs. Serous	2.667 (1.739-4.088)	<0.001	1.207 (0.533-2.735)	0.652
Histologic grade				
G1 vs. G2	7.117 (1.617-31.326)	0.009	8.736 (1.088-70.150)	0.041
G1 vs. G3	13.241 (3.247-53.993)	<0.001	7.016 (0.876-56.189)	0.066
Age (<=60 vs. >60)	1.847 (1.160-2.940)	0.010	2.071 (0.952-4.502)	0.066
Tumor invasion (%)	1.012 (1.008-1.016)	<0.001	1.006 (0.998-1.014)	0.167
Residual tumor (R0 vs. R2)	5.527 (2.879-10.612)	<0.001	2.857 (0.843-9.683)	0.092
Radiation tderapy (No vs. Yes)	0.594 (0.385-0.915)	0.018	0.344 (0.167-0.707)	0.004
EXO1 (Low vs. High)	1.761 (1.161-2.671)	0.008	1.402 (0.745-2.638)	0.294
THCA				
Patdologic stage				
Stage I vs. Stage III	9.733 (2.018-46.944)	0.005	7.390 (0.782-69.860)	0.081
Stage I vs. Stage IV	18.760 (3.601-97.751)	<0.001	18.800 (1.563-226.139)	0.021
Residual tumor (R0 vs. R1)	4.033 (1.214-13.402)	0.023	5.612 (1.142-27.574)	0.034
OV				
FIGO stage (Stage I&Stage II vs. Stage IV)	2.495 (1.057-5.889)	0.037	1.753 (0.399-7.709)	0.457
Primary tderapy outcome				
PD vs. SD	0.441 (0.217-0.895)	0.023	0.579 (0.269-1.246)	0.162
PD vs. CR	0.152 (0.093-0.247)	<0.001	0.209 (0.119-0.367)	<0.001
ERBB2 (Low vs. High)	1.505 (1.160-1.952)	0.002	1.299 (0.927-1.821)	0.129
Age (<=60 vs. >60)	1.355 (1.046-1.754)	0.021	1.323 (0.950-1.843)	0.098
Tumor residual (NRD vs. RD)	2.313 (1.486-3.599)	<0.001	1.097 (0.636-1.892)	0.739
Tumor status (Tumor free vs. Witd tumor)	9.576 (4.476-20.486)	<0.001	17.564 (4.270-72.248)	<0.001
CESC				
T stage (T1 vs. T3&T4)	4.019 (2.072-7.797)	<0.001	4736 (542 –41347)	<0.001
N stage (N0 vs. N1)	2.844 (1.446-5.593)	0.002	0.028 (0.006-0.131)	<0.001
TP53	0.757 (0.564-1.018)	0.065	0.097 (0.054-0.173)	<0.001
Clinical stage (Stage I vs. Stage IV)	4.376 (2.354-8.137)	<0.001	1541253 (0.000-Inf)	1.000
Primary tderapy outcome (PD vs. CR)	0.042 (0.022-0.082)	<0.001	0.000 (0.000-0.004)	<0.001
UCS				
Clinical stage				
Stage I vs. Stage II&Stage III	2.354 (1.020-5.435)	0.045	0.109 (0.003-4.086)	0.231
Stage I vs. Stage IV	2.817 (1.006-7.891)	0.049	0.000 (0.000-Inf)	0.999
Primary tderapy outcome (PD vs. SD&PR&CR)	0.105 (0.042-0.265)	<0.001	0.010 (0.000-0.422)	0.016
Histological type				
Heterologous Type vs. Homologous Type	0.261 (0.094-0.723)	0.010	0.473 (0.062-3.593)	0.469
Heterologous Type vs. NOS	0.461 (0.219-0.971)	0.042	0.183 (0.026-1.296)	0.089
Radiation tderapy (No vs. Yes)	0.404 (0.197-0.829)	0.013	9.174 (0.571-147.294)	0.118
Peritoneal wash (Negative vs. Positive)	3.460 (1.415-8.459)	0.007	6.150 (0.826-45.820)	0.076

### 3.4 Development of a prognostic model based on *EXO1* and clinical factors

**Figure 4:**
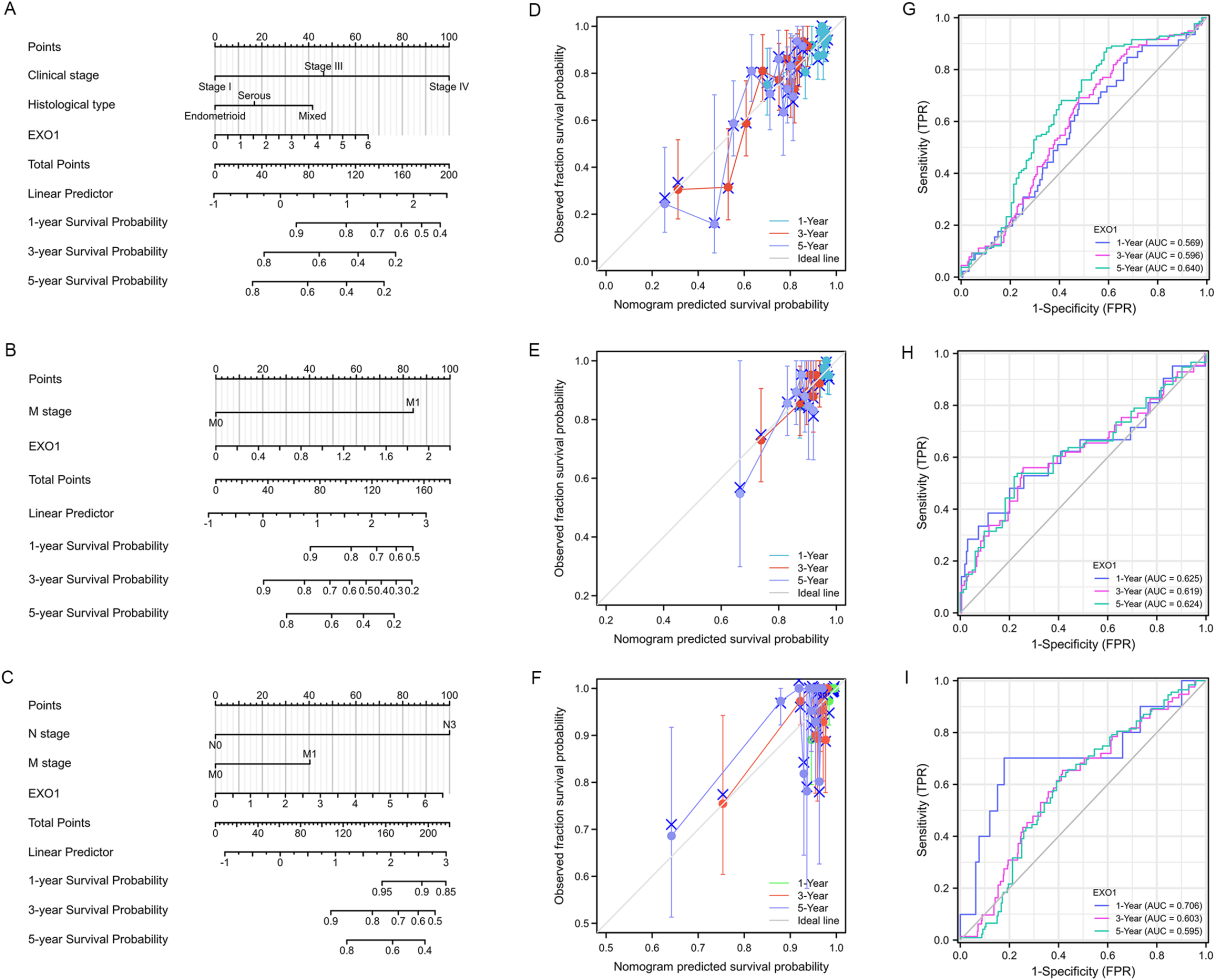
Construction of prognostic model based on *EXO1* expression and clinical factors for patients with female-related cancers. **(A–C)** Nomogram to predict the probability of 1-, 3-, and 5-year PFI in patients with UCEC **(A)** and THCA **(B)**, and DSS in patients with BRCA **(C)**. **(D–F)** Calibration plots of the nomogram of PFI in patients with UCEC **(D)** and THCA **(E)**, and DSS in patients with BRCA **(F)**. **(G–I)** AUC of time-dependent ROC curves verified the prognostic performance of EXO1 expression in TCGA. The abscissa is the false positive rate and the ordinate is the true positive rate. AUC, area under the curve; BRCA, breast cancer; DSS, disease-specific survival; *EXO1*, exonuclease 1; PFI, progression-free interval; ROC, receiver operating characteristic; TCGA, The Cancer Genome Atlas; THCA, thyroid cancer; UCEC, uterine corpus endometrial cancer.

To improve the prognostic prediction of patient outcomes, nomograms were constructed as prediction models by integrating *EXO1* expression and other significant clinical parameters determined by multivariate Cox analysis for PFI in UCEC (**Figure 4A**), THCA (**Figure 4B**), and DSS in BRCA (**Figure 4C**). The concordance-index values for the nomograms were 0.688, 0.648, and 0.721 for UCEC, THCA, and BRCA, respectively, indicating good agreement between the predicted outcomes and observed outcomes (**Figures 4D–F**). Additionally, the predictive performance of *EXO1* expression was assessed using time-dependent ROC curves. In UCEC, the AUC values for predicting PFI were 0.569, 0.596, and 0.640 at 1, 3, and 5 years, respectively (**Figures 4G**). In THCA, the AUC values for predicting PFI were 0.625, 0.619, and 0.624 at 1, 3, and 5 years, respectively (**Figure 4H**). Moreover, in BRCA, the AUC values for predicting DSS were 0.706, 0.603, and 0.595 at 1, 3, and 5 years, respectively (**Figure 4I**). These findings suggest that the nomograms incorporating *EXO1* expression may serve as useful models for predicting survival outcomes in UCEC, THCA, and BRCA.

### 3.5 Correlation between *EXO1* mutation and prognosis in female-related cancers

**Figure 5:**
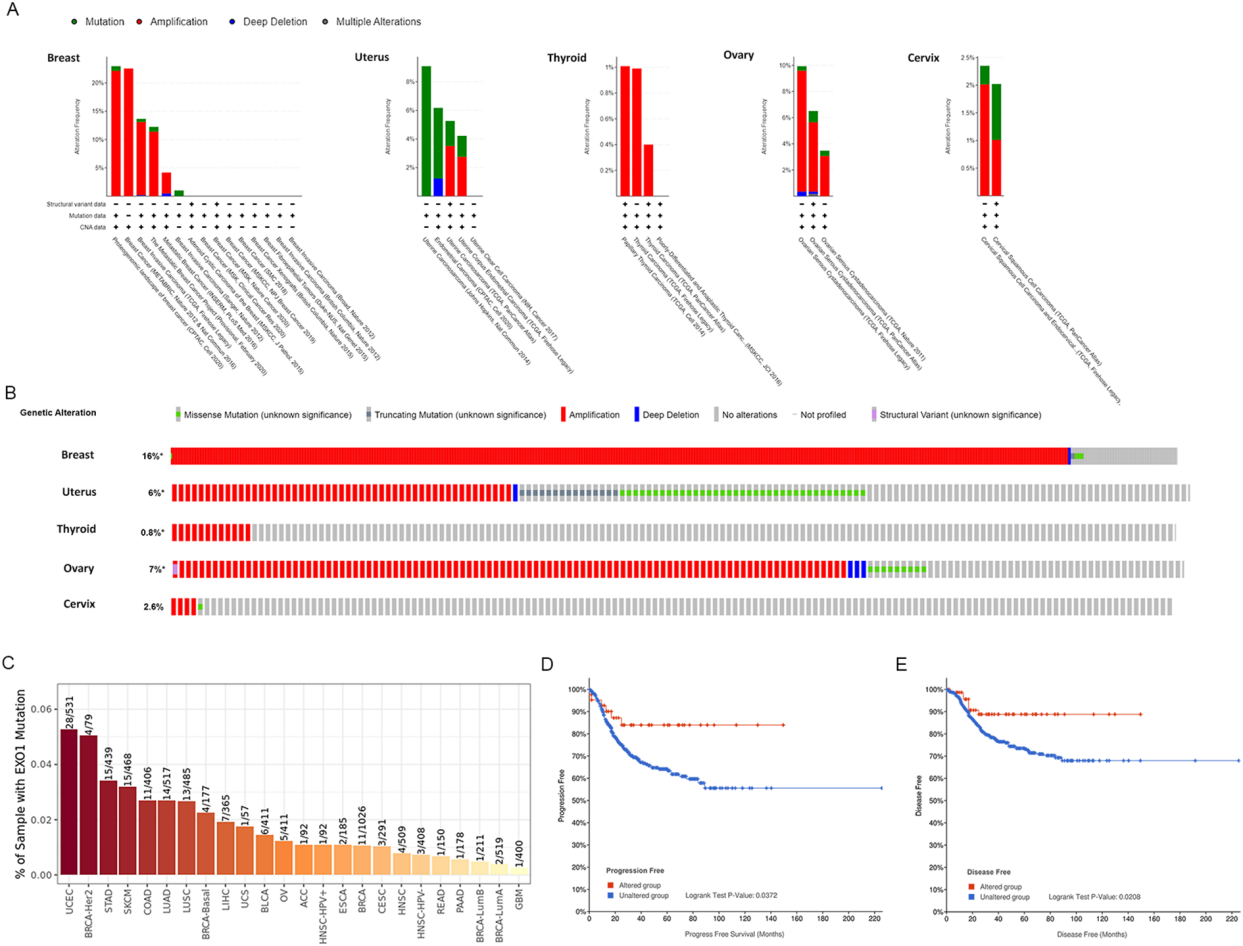
Alteration of *EXO1* gene in female-related cancers. **(A)** Mutation pattern analyses of *EXO1* with female-related cancer studies using cBioPortal. **(B)** OncoPrint visual summary of the alteration in *EXO1* gene with female-related cancers. **(C)** Genetic alteration analyses in *EXO1* in a pan-cancer dataset using TIMER2.0. **(D, E)** Genetic alteration in *EXO1* and its association with progression-free survival **(D)** and disease-free survival **(E)** of patients with UCEC. *EXO1*, exonuclease 1; UCEC, uterine corpus endometrial cancer.

After confirming the prognostic value of *EXO1*, we utilized cBioPortal and TIMER 2.0 to analyze the genetic alterations in *EXO1* across various female-related cancers. Analysis using the cBioPortal online tool revealed the presence of two or more alterations in *EXO1* in different female-related cancers, with amplification and deep deletion alterations being more common in uterine, breast, thyroid, and OV samples (**Figure 5A**). The percentage of *EXO1* gene alterations was 16% in BRCA samples, 6% in uterus cancer samples, 0.8% in THCA samples, 7% in OV samples and 2.6% in cervix cancer samples (**Figure 5B**). Results from TIMER 2.0 database demonstrated that among various types of cancers, UCEC had the highest mutation frequency for *EXO1*, with 28/531 cases showing mutations. The mutation frequencies for other cancers were as follows: 1/57, 5/411, and 3/291 cases in UCS, OV, and CESC, respectively (**Figure 5C**). In BRCA, the mutation frequency for *EXO1* was 11/1,026 cases. Further analysis based on molecular subtypes revealed the following mutation frequencies: 4/177, 4/79, 2/519, and 1/211 cases in basal-like BRCA, BRCA-HER2, luminal A BRCA, and luminal B BRCA, respectively (**Figure 5C**). Subsequently, we assessed the genetic alterations in *EXO1* and their associations with the prognosis of patients with female-related cancer. Our findings indicated that genetic alterations in *EXO1* were associated with improved progression-free survival and disease-free survival in patients with UCEC (**Figures 5D, E**). These results suggest that the genetic mutations of *EXO1* also impact the prognosis of patients with UCEC.

### 3.6 Correlation between *EXO1* methylation status and prognosis in female-related cancers

**Figure 6:**
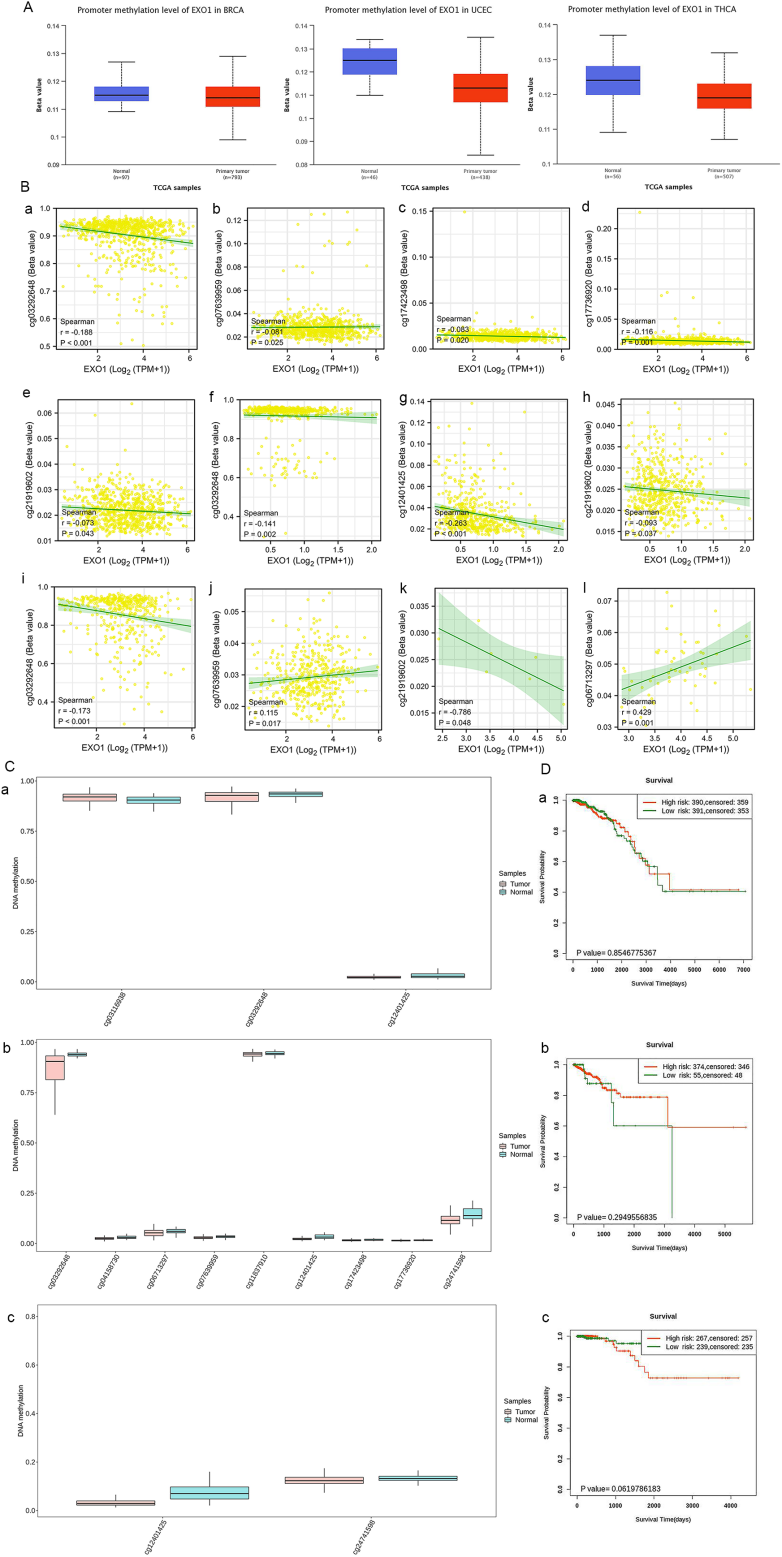
Methylation levels of *EXO1* in patients with female-related cancers. **(A)** The promoter methylation of *EXO1* in normal tissues and tumor tissues from TCGA data in UALCAN. **(B)** Cor-relation of DNA methylation and *EXO1* expression at multiple probes in female-related cancers, including BRCA (a–e), THCA (f–h), UCEC (i, j), OV (k), and UCS (l). **(C)** Analyses of methylation differences in multiple probes between tumor and normal groups in BRCA (a), UCEC (b), THCA (c) using SurvivalMeth. **(D)** Kaplan–Meier survival plots constructed in multiple probes between high- and low-risk groups in BRCA (a), UCEC (b), and THCA (c) using SurvivalMeth. BRCA, breast cancer; *EXO1*, exonuclease 1; OV, ovarian cancer; TCGA, The Cancer Genome Atlas; THCA, thyroid cancer; UCEC, uterine corpus endometrial cancer; UCS, uterine sarcoma.

After confirming the presence of *EXO1* alterations, we investigated the association between mRNA expression of *EXO1* and its methylation status in selected cancers. Analysis of data from TCGA database through the UALCAN webpage revealed that the promoter methylation level of *EXO1* was significantly lower in tumor tissues compared to normal tissues in various cancers, including BRCA, UCEC, and THCA (**Figure 6A**). Further analysis was conducted to examine the relationship between *EXO1* methylation and gene expression. The results showed negative correlation between the methylation of five specific CpG sites (cg03292648, cg07639959, cg17423498, cg17736920, cg21919602) and EXO1 gene expression in BRCA, three CpG sites (cg03292648, cg12401425, cg21919602) and EXO1 gene expression in THCA; one CpG site (cg03292648) negatively and one CpG site (cg06713297) positively correlated with *EXO1* gene expression in UCEC; one CpG site (cg21919602) negatively correlated with *EXO1* gene expression in OV; one CpG site (cg06713297) positively correlated with *EXO1* gene expression in UCS (**Figure 6B**). Additionally, using the SurvivalMeth tool, significant differences in the expression of individual CpG site methylation of *EXO1* were observed between tumor tissues and normal tissues in BRCA, UCEC, and THCA (**Figure 6C**). However, no significant prognostic correlation was found between high- and low-risk groups in female-related cancers (**Figure 6D**). These findings suggest that DNA methylation may be associated with *EXO1* expression, but may not be related to the prognosis in female-related cancers.

### 3.7 Function and pathway enrichment analysis of *EXO1* in female-related cancers

**Figure 7:**
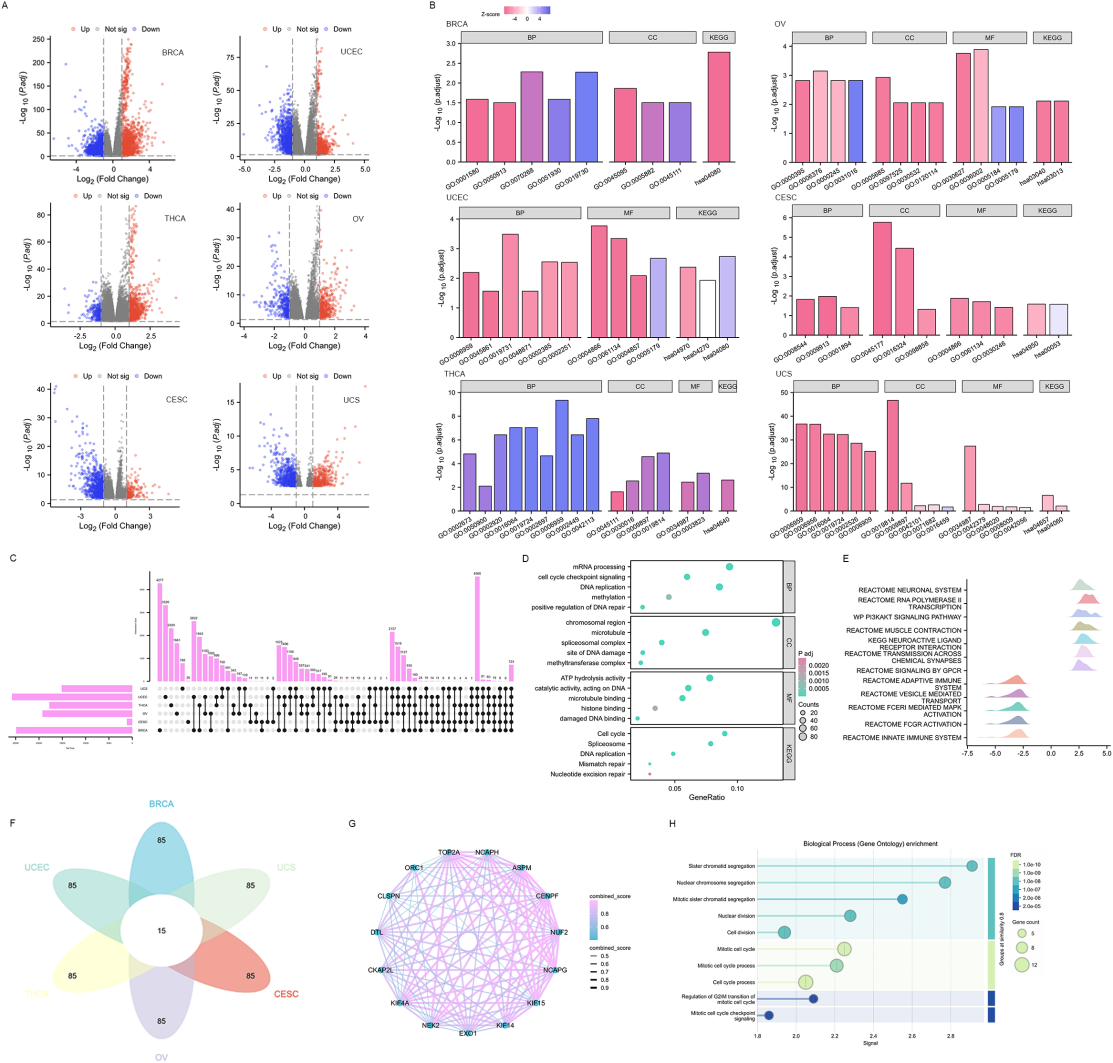
Functional enrichment analysis of DEGs in female-related cancers. **(A)** Volcano plots of total DEGs between high and low *EXO1* expression groups. Red and green plots indicate upregulated and downregulated genes, respectively, and the black plots show those with differential expression below the cutoff criteria. **(B)** Enriched GO terms in the “biological process” category, “cellular component” category, “molecular function” category, and KEGG classification of DEGs. **(C)** Upset diagram demonstrating the overlap between genes correlated with EXO1 expression among female-related cancers. **(D)** Enriched GO terms and KEGG classification of genes correlated with EXO1 expression. **(E)** Ridgeline plot of Gene Set Enrichment Analysis (GSEA) for *EXO1*-correlated genes. **(F)** Flower plot illustrating the intersection of top 100 *EXO1*-associated genes across six cancer types. **(G)** Protein-protein interaction (PPI) network of 15 co-correlated genes. Nodes represent genes, with edge thickness proportional to interaction confidence scores. **(H)** Functional enrichment analysis of the PPI network for biological processes (Gene Ontology). DEGs, differentially expressed genes; *EXO1*, exonuclease 1; GO, Gene Ontology; KEGG, Kyoto Encyclopedia of Genes and Genomes.

DEGs were identified between high and low *EXO1* expression groups in different female-related cancers. The analysis revealed 629 DEGs (274 upregulated and 355 downregulated) in BRCA and 120 DEGs (37 upregulated and 83 downregulated) in UCEC, and fewer DEGs in THCA, OV, CESC, and UCS (**Figure 7A** and **Supplementary Table S3**). Functional enrichment analysis showed that the DEGs were involved in processes related to the regulation of cell differentiation, regulation of immune/inflammatory response, as well as chemokine and chemokine receptor activity. KEGG pathway enrichment analysis revealed that the DEGs were mainly associated with cell cycle, DNA replication, DNA repair mechanisms, interleukin 17 (IL17) signaling pathway, and cytokine-cytokine receptor interaction (**Figure 7B** and **Supplementary Table S4**). Furthermore, a total of 723 *EXO1*-correlated genes were identified in all cancer types (**Figure 7C**). These genes were enriched in cell cycle regulation, DNA repair (replication, mismatch/nucleotide excision repair), and epigenetic modulation (methylation, methyl-transferase complexes) via molecular functions such as DNA/microtubule/histone binding and ATP-dependent catalysis (**Figure 7D**). GSEA pathway analysis further indicated that high EXO1 expression was upregulated in pathways related to the phosphatidylinositol 3 kinase-protein kinase B (PI3K-AKT) signaling pathway, muscle contraction, and transmission across chemical synapses. Meanwhile, pathways such as signaling by G protein-coupled receptor (GPCR), adaptive immune system, vesicle-mediated transport, Fc epsilon receptor I-mediated (FCER I-mediated) mitogen-activated protein kinase (MAPK) activation, Fc gamma receptor (FCGR) activation, and innate immune system were downregulated (**Figure 7E**). Based on the correlation strength, we identified the top 100 genes significantly associated with *EXO1* in each of the six cancer types and determined their intersection, yielding 15 genes that exhibited consistent high correlation with *EXO1* across all six cancers (**Figure 7F**). The STRING-based protein interaction network of EXO1 (high-confidence score ≥0.7) revealed a tightly clustered module with 15 nodes and 98 edges, demonstrating exceptionally significant interaction enrichment (**Figure 7G**). Functional annotation highlighted EXO1’s predominant involvement in mitotic regulation and chromosome dynamics (**Figure 7H**). These pathways are frequently associated with oncogenesis and immune-related processes, including immunotherapy and cell chemotaxis.

### 3.8 Correlations between *EXO1* expression and tumor immune infiltration cells

**Figure 8:**
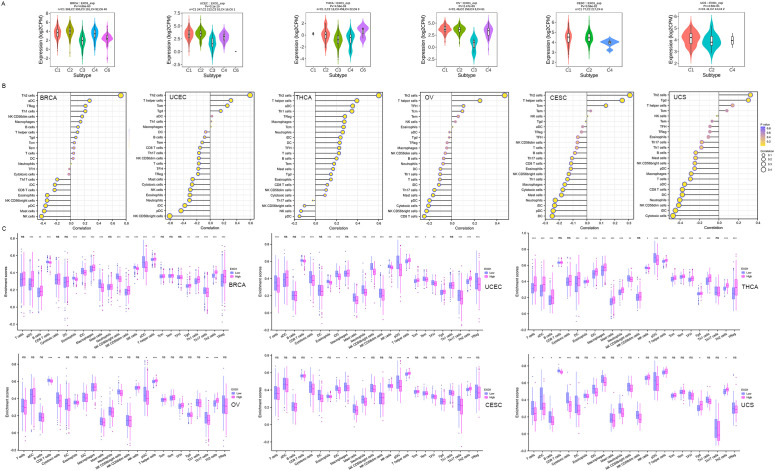
Correlation between *EXO1* expression and TIICs into the UCEC tumor microenvironment. **(A)** Correlations between *EXO1* expression and immune cell subtypes in female-related cancers from TISIDB database. C1: wound healing, C2: IFNG dominant, C3: inflammatory, C4: lymphocyte depleted, C5: immunologically quiet, C6: TGFB dominant. **(B)** Spearman correlation analysis between *EXO1* expression levels and infiltration levels of 24 indicated immune cell types in tumor tissues. Dot size indicates the absolute Spearman correlation coefficient; and the color gradation from blue to yellow indicates high to low *P*-values. **(C)** Comparisons of TIIC infiltration levels between the high and low *EXO1* expression groups. *EXO1*, exonuclease 1; IFNG, interferon-gamma; TGFB, transforming growth factor beta; TIIC, tumor immune infiltration cell; UCEC, uterine corpus endometrial cancer. ns: P ≥ 0.05; *P < 0.05; **P < 0.01; ***P < 0.001.

We investigated the relationship between *EXO1* expression and immune cell subtypes in female-related cancers using the TISIDB database. Our analysis revealed significantly associations between *EXO1* expression and different immune cell subtypes in BRCA, UCEC, THCA, OV, and CESC (**Figure 8A**). Furthermore, we explored the correlations between *EXO1* expression and the infiltration of 24 types of TIICs into the tumor microenvironment. The results revealed that *EXO1* expression is positively correlated with the infiltration of T helper 2 (Th2) cells and T helper cells, and negatively correlated with natural killer (NK) CD56bright cells and plasmacytoid dendritic cells (pDC) in all six cancer types (**Figure 8B**). Our analysis also revealed cancer subtype-specific patterns, in BRCA and THCA, *EXO1* showed positive associations with regulatory T cells (Tregs) and activated dendritic cells (aDC), whereas UCEC and OV exhibited inverse correlations with cytotoxic CD8+ T cells. In CESC and UCS, *EXO1* exhibited mixed immunomodulatory profiles involving positive correlations with T helper cells, alongside broad negative associations with cytotoxic populations and dendritic cell subsets (detailed in **Figure 8B** and **Supplementary Table S5**). We further compared the enrichment scores of immune cells in the high and low *EXO1* expression groups. The results consistently showed that the infiltration levels of Th2 cells were higher in the high *EXO1* expression group across all six cancer types (**Figure 8C**). In summary, our findings suggest that high *EXO1* expression is associated with increased infiltration of Th2 cells in female-related cancers.

### 3.9 *EXO1* modulates immune regulatory networks and therapeutic responses

**Figure 9:**
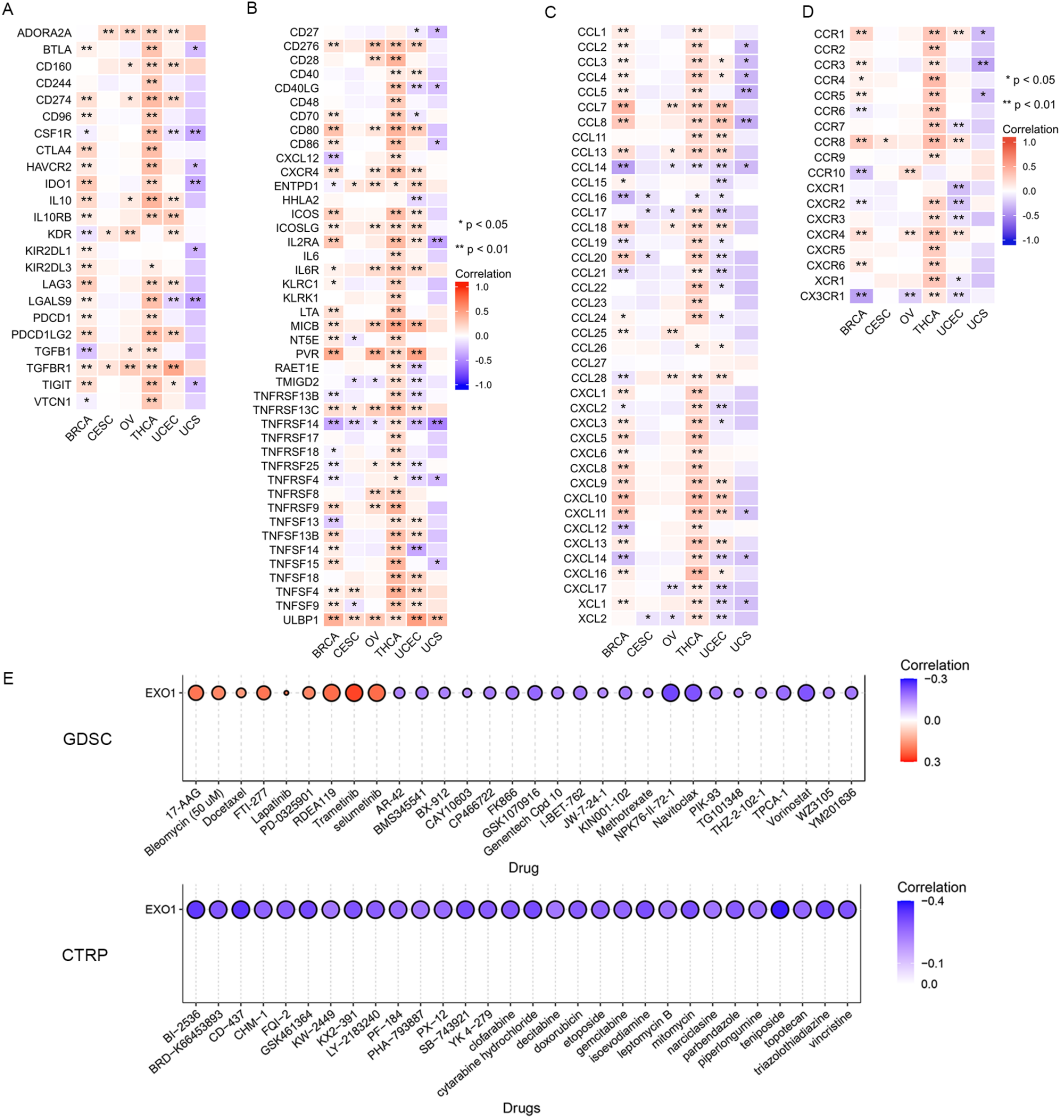
Correlation between *EXO1* expression and immune inhibitors **(A)**, immune stimulators **(B)** chemokines **(C)**, and chemokine receptors **(D)** in female-related cancers. **(E)** The correlation between *EXO1* expression and the sensitivity to GDSC and CTRP drugs (top 30) in a pan-cancer dataset. ns: *P* ≥ 0.05; *P < 0.05; **P < 0.01. CTRP, Cancer Therapeutics Response Portal; *EXO1*, exonuclease 1; GDSC, Genomics of Drug Sensitivity of Cancer; ns, no significance.

We further investigated the associations between *EXO1* and several well-established immune checkpoint genes. Our analysis revealed that *EXO1* expression was broadly correlated with immune regulatory molecules across female-related cancers. It showed positive associations with inhibitory checkpoints such as programmed cell death 1 (*PDCD1*), cytotoxic T-lymphocyte associated protein 4 (*CTLA4*), and transforming growth factor beta receptor 1 (*TGFBR1*), as well as immune stimulators like *CD40* and C-X-C motif chemokine receptor 4 (*CXCR4*). However, in UCS, EXO1 expression was inversely related to T cell immunoreceptor with Ig and ITIM domains (*TIGIT*) and colony stimulating factor 1 receptor (*CSF1R*) (**Figures 9A, B**; **Supplementary Table S6**). Cell chemotaxis plays a role in functional enrichment, and chemokines and chemokine receptors mediate the movement of tumor cells and immune cells. Thus, we explored the correlation between *EXO1* and chemokines/chemokine receptors in female-related cancers. Our analysis showed two prominent patterns. One pattern was a Th2/chemotaxis axis characterized by strong positive correlations with C-C motif chemokine receptor 8 (*CCR8*), *CXCR4*, and Th2-associated chemokines such as C-C motif chemokine ligand 7 (*CCL7*), *CCL13* and *CCL18*. The other pattern was characterized by inflammatory suppression marked by negative links to C-X-3-C motif chemokine receptor 1 (*CX3CR1*) and pro-inflammatory chemokines (*CCL14/16*) (**Figures 9C, D**; **Supplementary Table S7**).

Lastly, we investigated the correlation between *EXO1* expression and drug sensitivity in cancers. We found high *EXO1* expression predicted sensitivity to MEK inhibitors (trametinib, selumetinib) and resistance to HDAC inhibitors (vorinostat) and apoptosis-targeting agents (navitoclax) (**Figure 9E**). These findings position *EXO1* as a potential biomarker for Th2-polarized immune microenvironments and a predictor of therapeutic vulnerability in female-related cancers.

## 4 Discussion

DNA damage and repair signaling pathways have been shown to play a critical role in maintaining genomic stability. Defects in these pathways can have dual effects, contributing to tumorigenesis while also rendering cancer cells vulnerable to DNA damage and reliant on DNA repair and signaling activity (30). *EXO1* has been implicated in multiple DNA repair pathways that safeguard DNA replication, including mismatch repair, translation synthesis, homologous recombination, and cell cycle regulation (14). Given its central role in replication and post-replication processes, including checkpoint activation, it is likely that dysfunction of *EXO1* may perturb chromosomal stability and disrupt other DNA repair pathways. This can lead to replication stress, translocations, transformation, and cell death, ultimately resulting in genomic instability and the development of cancer (31).

Several lines of evidence suggest that *EXO1* is overexpressed in certain tumor cells and may serve as a candidate susceptibility gene for breast, ovarian, lung, and gastrointestinal cancers. It also holds potential as a target for cancer therapy due to its increased expression in tumors (32–36). However, the precise role of *EXO1* in the development of various female-related cancers, particularly through the DNA damage and repair signaling pathway, remains unknown. Therefore, the aim of this study was to provide an overview of the *EXO1* gene in multiple female-related cancers. We conducted a comprehensive evaluation of the potential roles of *EXO1* in different female-related cancers using RNA-seq data from TCGA database. This analysis included *EXO1* gene expression patterns, prognostic values, genetic mutations, DNA methylation, immune infiltration, gene interactions, and drug sensitivities. By exploring these aspects, we sought to shed light on the broader landscape of *EXO1* in female-related cancers and unravel its potential implications for disease progression and treatment response.

Our study demonstrated high *EXO1* expression in multiple female-related cancers, including BRCA, CESC, OV, THCA, UCEC, and UCS, compared to healthy controls. This finding aligns with previous studies that have also reported overexpression of *EXO1* in various cancer types, such as breast, ovarian, lung, liver, prostate, bladder, and melanoma (35, 37–45). In addition to its overexpression in tumor tissues, we observed that *EXO1* showed a strong discriminatory ability between female-related tumors and normal tissues, except UCS. Given that *EXO1* plays a crucial role in regulating the cell cycle and DNA repair processes (46, 47), it is expected to be upregulated in proliferating cells, particularly in cancer cells characterized by an active cell cycle and high proliferation rate. Therefore, the higher expression of *EXO1* in tumor tissues is consistent with this expectation.

In addition, we observed significant and distinct associations between *EXO1* expression levels and clinicopathological characteristics in the six aforementioned female-related cancers. For example, *EXO1* was found to be expressed at higher levels in Asians compared to White patients with BRCA and CESC. It was also higher in patients with UCEC and UCS who received radiation therapy. Moreover, *EXO1* expression was elevated in pathologic stage II of BRCA, FIGO stage I–II of OV, and histologic grade 3 of UCEC and CESC. Additionally, in BRCA and CESC, *EXO1* expression was higher in patients aged <60 years, whereas in UCEC, it was higher in patients aged >60 years. These findings indicate that, although *EXO1* expression is increased in various female-related cancers, there may be distinct patterns of expression across different cancer types.

Furthermore, we conducted Kaplan-Meier survival analysis to evaluate the prognostic value of *EXO1* in female-related cancers. High *EXO1* expression was significantly associated with worse OS, PFI, and DSS in BRCA and UCEC, and correlated with worse PFI in THCA. Moreover, considering the different associations between *EXO1* expression and clinicopathological characteristics, we analyzed the correlations between *EXO1* expression and prognosis in different subgroups based on clinical parameters in patients with female-related cancers. The results suggested that higher *EXO1* expression was generally associated with worse OS across most clinical subgroups. However, in specific subgroups, such as the basal type of PAM50 classification in BRCA, T2 stage or squamous cell carcinoma in CESC, and FIGO stage IV in OV, higher *EXO1* expression was associated with better OS. We hypothesized that this could be related to increased DNA repair capacity mediated by *EXO1*, possibly due to lymphocyte infiltration or invasion of adjacent organs in these subgroups. Moreover, through univariate and multivariate Cox regression analyses, we identified clinical stage, primary therapy outcome, histological type, residual tumor, and *EXO1* expression levels as independent risk factors for PFI in UCEC, M stage, and *EXO1* expression level as independent risk factors for PFI in THCA, and N stage, M stage, and *EXO1* expression levels as independent risk factors for DSS in BRCA. Based on these findings, we constructed nomograms that integrated *EXO1* expression and these clinical parameters to predict PFI in UCEC and THCA, and DSS in BRCA. These results are consistent with those of previous studies that have also identified *EXO1* as a potential prognostic marker in BRCA, OV, lung cancer, and liver cancer (38). Our data further support the notion that *EXO1* has predictive and prognostic value and may serve as a biomarker in certain female-related cancers.

Previous reports have suggested that mutations in the *EXO1* gene can lead to protein dysfunction and increased susceptibility to certain cancers (48). In our genetic mutation analysis, we observed a higher frequency of *EXO1* mutations in UCEC and BRCA compared to other female-related cancers. Interestingly, *EXO1* mutation was associated with a better prognosis in UCEC, but did not show a significant association with prognosis in the other five female-related cancers. Additionally, DNA methylation can influence gene expression levels and impact patient prognosis. Our study revealed that DNA methylation levels in the *EXO1* gene were significantly lower in tumor tissues compared to normal tissues in BRCA, UCEC, and THCA. This result is consistent with our findings of upregulated *EXO1* expression in those cancers. However, the DNA methylation levels of *EXO1* did not show a significant prognostic correlation in the female-related cancers we analyzed. Based on these observations, we speculate that gene alterations and methylation changes in *EXO1* may not be the main driving factors in the development of female-related cancers or affecting patient prognosis. Other mechanisms and factors are likely involved in the pathogenesis and prognosis of these cancer types. Further studies are needed to explore additional molecular and biological processes that contribute to the development and progression of female-related cancers.

To further investigate the biological function of *EXO1* in female-related cancers, we conducted differential gene expression analysis between high and low *EXO1* expression groups, as well as correlation analysis of *EXO1* with other expressed genes using TCGA data. We identified hundreds of DEGs and correlated genes, which we further subjected to functional enrichment analysis. The results of the functional enrichment analysis revealed that the DEGs were associated with various pathways involved in cell cycle, oncogenesis, and immune-related processes in female-related cancers. These pathways included DNA replication, DNA repair, regulation of cell differentiation, inflammatory response, chemokine and chemokine receptor activity, PI3K-AKT signaling pathway, signaling by GPCR, FCER I-mediated MAPK activation, and FCGR activation. To evaluate the relationship between *EXO1* expression and immune-related factors, we examined the associations between *EXO1* expression and immune inhibitors, immune stimulators, chemokines, and chemokine receptors in female-related cancers. Interestingly, we found that in UCS, *EXO1* was negatively associated with certain immune inhibitors, immune stimulators, chemokines, and chemokine receptors. However, in BRCA, OV, THCA, and UCEC, *EXO1* was positively associated with most of these factors. From another perspective, we observed significant positive correlations between *EXO1* expression and *TGFBR1* and *ULBP1* in most female-related cancers, while a negative correlation was found with *TNFRSF14* (herpesvirus entry mediator). Previous studies have implicated *TGFBR1* polymorphic variants in bladder cancer risk and prognosis (34, 49). Moreover, *ULBP1* has been shown to promote immune escape via *PDCD1LG1* in hepatocellular carcinoma (HCC) (50). Furthermore, increased expression of *TNFRSF14* has been associated with favorable prognosis in bladder cancer and BRCA (51, 52). Taken together, these findings suggest that these molecules serve as potential targets for *EXO1* in different female-related cancers.

Additionally, our analysis of chemokines revealed a strong positive correlation between *EXO1* expression and *CCL7*, *CCL13*, and *CCL18* in BRCA, OV, THCA, and UCEC, while a negative correlation was observed with *CCL14* and *CCL16* in most female-related cancers. In terms of chemokine receptors, *EXO1* showed a high positive correlation with *CCR8* and *CXCR4*, while a negative correlation was found with *CX3CR1* in most female-related cancers. These observations are in line with previous reports highlighting the crucial roles of CC chemokines in immune cell functioning (53). For example, *CCL18* has been shown to promote the proliferation of oral squamous cell carcinoma cells and recruit naive CD4+ T cells into the tumor microenvironment, leading to their differentiation into regulatory T cells that contribute to tumor immune evasion (54, 55). *CCL14* has been associated with suppression of HCC progression, promotion of HCC cell apoptosis, and longer OS in patients with HCC (56). Increased *CCR8* expression has been observed in tumor cells of malignant melanoma and is involved in metastasis to peripheral lymphoid organs (57). These results suggest a potential role of *EXO1* in the development and modulation of the tumor immune microenvironment in female-related cancers through its association with these chemokines and receptors.

To further explore the association between *EXO1* and cancer immunity, we investigated the relationship between *EXO1* expression and different immune subtypes using the TISIDB database. Our findings indicated that *EXO1* expression was significantly associated with immune subtypes in five of the six female-related cancers analyzed, showing higher expression in C1 (wound healing) and C2 (interferon-gamma [IFNG] dominant) subtypes, but lower expression in C3 (inflammatory) subtypes. Notably, our analysis of immune infiltration revealed that *EXO1* expression was positively correlated with the infiltration of Th2 cells and Th cells, while it was negatively correlated with NK CD56bright cells and pDC. Moreover, we consistently observed higher infiltration levels of Th2 cells in the high *EXO1* expression groups across all six cancer types. Th2 cells are elevated in human cancers and secrete various effector cytokines, such as *IL4*, *IL5*, *IL6*, *IL10*, and *IL13* (58, 59). Patients with a dominant Th2 response in the tumor microenvironment have been associated with poorer prognosis (13). In our study, the increased infiltration of Th2 cells in the context of *EXO1* overexpression may contribute to an imbalance between Th1 and Th2 responses, enabling tumor cells to evade immunity. Furthermore, it has been reported that *EXO1* overexpression leads to indefinite DNA excision, resulting in chromosomal abnormalities and the release of nuclear DNA into the cytoplasm. This activates the cyclic GMP-AMP synthase-stimulator of interferon response cGAMP interactor (cGAS-STING) pathway, which plays a critical regulatory role in tumor immunity (60).

In this study, through bioinformatics analyses, we demonstrated that *EXO1* is closely associated with immune cell infiltration and immune molecule expression, suggesting its potential as a predictor of prognosis and response to immunotherapy in patients with various female-related cancers. Finally, our study revealed that *EXO1* may be associated with the sensitivity to multiple anticancer drugs that inhibit proliferation or interfere with the cell cycle of cancer cells. This implies that *EXO1* may serve as a biomarker for predicting drug sensitivity.

## 5 Conclusions

Collectively, the present data indicate that *EXO1* may play a role in the development and therapeutic response of female-related cancers by affecting multiple pathways, such as DNA repair, cell cycle regulation, immune microenvironmental regulation, and chemotherapeutic drug sensitivity.

## Data Availability

The original contributions presented in the study are included in the article/**Supplementary Material**. Further inquiries can be directed to the corresponding author.

## References

[1] Ferlay J Colombet M Soerjomataram I Parkin DM Piñeros M Znaor A. Cancer statistics for the year 2020: An overview. Int J Cancer. (2021) 149:778–89. doi: 10.1002/ijc.v149.4 33818764

[2] Siegel RL Giaquinto AN Jemal A. Cancer statistics, 2024. CA Cancer J Clin. (2024) 74:12–49. doi: 10.3322/caac.21820 38230766

[3] Chen W Zheng R Baade PD Zhang S Zeng H Bray F. Cancer statistics in China, 2015. CA Cancer J Clin. (2016) 66:115–32. doi: 10.3322/caac.21338 26808342

[4] Lin S Gao K Gu S You L Qian S Tang M. Worldwide trends in cervical cancer incidence and mor-tality, with predictions for the next 15 years. Cancer. (2021) 127:4030–9. doi: 10.1002/cncr.v127.21 34368955

[5] Siegel RL Miller KD Jemal A. Cancer statistics, 2020. CA Cancer J Clin. (2020) 70:7–30. doi: 10.3322/caac.21590 31912902

[6] Huang J Chan WC Ngai CH Lok V Zhang L Lucero-Prisno DE 3rd. Worldwide burden, risk factors, and temporal trends of ovarian cancer: A global study. Cancers (Basel). (2022) 14:2230. doi: 10.3390/cancers14092230 35565359 PMC9102475

[7] Xia C Dong X Li H Cao M Sun D He S. Cancer statistics in China and United States, 2022: profiles, trends, and determinants. Chin Med J (Engl). (2022) 135:584–90. doi: 10.1097/CM9.0000000000002108 PMC8920425 35143424

[8] Shank JB Are C Wenos CD. Thyroid cancer: global burden and trends. Indian J Surg Oncol. (2022) 13:40–5. doi: 10.1007/s13193-021-01429-y PMC8986939 35462648

[9] Shobab L Burman KD Wartofsky L. Sex differences in differentiated thyroid cancer. Thyroid. (2022) 32:224–35. doi: 10.1089/thy.2021.0361 34969307

[10] El-Shemerly M Janscak P Hess D Jiricny J Ferrari S. Degradation of human exonuclease 1b upon DNA synthesis inhibition. Cancer Res. (2005) 65:3604–9. doi: 10.1158/0008-5472.CAN-04-4069 15867354

[11] Bolderson E Richard DJ Edelmann W Khanna KK. Involvement of Exo1b in DNA damage-induced apoptosis. Nucleic Ac-ids Res. (2009) 37:3452–63. doi: 10.1093/nar/gkp194 PMC2691832 19339515

[12] Keijzers G Bakula D Petr MA Madsen NGK Teklu A Mkrtchyan G. Human exonuclease 1 (EXO1) regulatory functions in DNA replication with putative roles in cancer. Int J Mol Sci. (2018) 20:74. doi: 10.3390/ijms20010074 30585186 PMC6337416

[13] Guan J Lu C Jin Q Lu H Chen X Tian L. MLH1 deficiency-triggered DNA hyperexcision by exonuclease 1 activates the cGAS-STING pathway. Cancer Cell. (2021) 39:109–121.e5. doi: 10.1016/j.ccell.2020.11.004 33338427 PMC8666006

[14] Sertic S Quadri R Lazzaro F Muzi-Falconi M. EXO1: A tightly regulated nuclease. DNA Repair (Amst). (2020) 93:102929. doi: 10.1016/j.dnarep.2020.102929 33087266

[15] Lawrenson K Iversen ES Tyrer J Weber RP Concannon P Hazelett DJ. Common variants at the CHEK2 gene locus and risk of epithelial ovarian cancer. Car-cinogenesis. (2015) 36:1341–53. doi: 10.1093/carcin/bgv138 PMC4635670 26424751

[16] Zhou J Wang Y Wang Y Yin X He Y Chen L. FOXM1 modulates cisplatin sensitivity by regulating EXO1 in ovarian cancer. PloS One. (2014) 9:e96989. doi: 10.1371/journal.pone.0096989 24824601 PMC4019642

[17] Wei K Clark AB Wong E Kane MF Mazur DJ Parris T. Inactivation of Exonuclease 1 in mice results in DNA mismatch repair defects, increased cancer susceptibility, and male and female sterility. Genes Dev. (2003) 17:603–14. doi: 10.1101/gad.1060603 PMC196005 12629043

[18] Vivian J Rao AA Nothaft FA Ketchum C Armstrong J Novak A. Toil enables reproducible, open source, big biomedical data analyses. Nat Biotechnol. (2017) 35:314–6. doi: 10.1038/nbt.3772 PMC5546205 28398314

[19] Tang Z Kang B Li C Chen T Zhang Z. GEPIA2: an enhanced web server for large-scale expression profiling and inter-active analysis. Nucleic Acids Res. (2019) 47:556–60. doi: 10.1093/nar/gkz430 PMC6602440 31114875

[20] Robin X Turck N Hainard A Tiberti N Lisacek F Sanchez JC. pROC: an open-source package for R and S+ to analyze and compare ROC curves. BMC Bioinf. (2011) 12:77. doi: 10.1186/1471-2105-12-77 PMC3068975 21414208

[21] Iasonos A Schrag D Raj GV Panageas KS. How to build and interpret a nomogram for cancer prognosis. J Clin Oncol. (2008) 26:1364–70. doi: 10.1200/JCO.2007.12.9791 18323559

[22] Liu J Lichtenberg T Hoadley KA Poisson LM Lazar AJ Cherniack AD. An integrated TCGA pan-cancer clinical data resource to drive high-quality survival outcome analytics. Cell. (2018) 173:400–416.e11. doi: 10.1016/j.cell.2018.02.052 29625055 PMC6066282

[23] Gao J Aksoy BA Dogrusoz U Dresdner G Gross B Sumer SO. Integrative analysis of complex cancer genomics and clinical profiles using the cBioPortal. Sci Signal. (2013) 6:pl1. doi: 10.1126/scisignal.2004088 23550210 PMC4160307

[24] Chandrashekar DS Bashel B Balasubramanya SAH Creighton CJ Ponce-Rodriguez I Chakravarthi BVSK. UALCAN: A portal for facilitating tumor subgroup gene expression and survival analyses. Neoplasia. (2017) 19:649–58. doi: 10.1016/j.neo.2017.05.002 PMC5516091 28732212

[25] Zhang C Zhao N Zhang X Xiao J Li J Lv D. SurvivalMeth: a web server to investigate the effect of DNA methylation-related functional elements on prognosis. Brief Bioinform. (2021) 22:bbaa162. doi: 10.1093/bib/bbaa162 32778890

[26] Love MI Huber W Anders S. Moderated estimation of fold change and dispersion for RNA-seq data with DESeq2. Genome Biol. (2014) 15:550. doi: 10.1186/s13059-014-0550-8 25516281 PMC4302049

[27] Yu G Wang LG Han Y He QY. clusterProfiler: an R package for comparing biological themes among gene clusters. OMICS. (2012) 16:284–7. doi: 10.1089/omi.2011.0118 PMC3339379 22455463

[28] Ru B Wong CN Tong Y Zhong JY Zhong SSW Wu WC. TISIDB: an integrated repository portal for tumor-immune system interactions. Bioinformatics. (2019) 35:4200–2. doi: 10.1093/bioinformatics/btz210 30903160

[29] Bindea G Mlecnik B Tosolini M Kirilovsky A Waldner M Obenauf AC. Spatiotemporal dynamics of intratumoral immune cells reveal the immune landscape in human cancer. Immunity. (2013) 39:782–95. doi: 10.1016/j.immuni.2013.10.003 24138885

[30] Hopkins JL Lan L Zou L. DNA repair defects in cancer and therapeutic opportunities. Genes Dev. (2022) 36:278–93. doi: 10.1101/gad.349431.122 PMC8973847 35318271

[31] Keijzers G Liu D Rasmussen LJ. Exonuclease 1 and its versatile roles in DNA repair. Crit Rev Biochem Mol Biol. (2016) 51:440–51. doi: 10.1080/10409238.2016.1215407 27494243

[32] Tsai MH Tseng HC Liu CS Chang CL Tsai CW Tsou YA. Interaction of Exo1 genotypes and smoking habit in oral cancer in Taiwan. Oral Oncol. (2009) 45:e90–4. doi: 10.1016/j.oraloncology.2009.03.011 19515603

[33] Wang HC Chiu CF Tsai RY Kuo YS Chen HS Wang RF. Association of genetic polymorphisms of EXO1 gene with risk of breast cancer in Taiwan. Anticancer Res. (2009) 29:3897–901. 19846925

[34] Zhang M Zhao D Yan C Zhang L Liang C. Associations between nine polymorphisms in EXO1 and cancer susceptibility: A systematic review and meta-analysis of 39 case-control studies. Sci Rep. (2016) 6:29270. doi: 10.1038/srep29270 27387683 PMC4937237

[35] Dai Y Tang Z Yang Z Zhang L Deng Q Zhang X. EXO1 overexpression is associated with poor prog-nosis of hepatocellular carcinoma patients. Cell Cycle. (2018) 17:2386–97. doi: 10.1080/15384101.2018.1534511 PMC6237436 30328366

[36] Muthuswami M Ramesh V Banerjee S Viveka Thangaraj S Periasamy J Bhaskar Rao D. Breast tumors with elevated expression of 1q candidate genes confer poor clinical outcome and sensitivity to Ras/PI3K inhibition. PloS One. (2013) 8:e77553. doi: 10.1371/journal.pone.0077553 24147022 PMC3798322

[37] Li Y Wang Y Zhang W Wang X Chen L Wang S. BKM120 sensitizes BRCA-proficient triple negative breast cancer cells to olaparib through regulating FOXM1 and Exo1 expression. Sci Rep. (2021) 11:4774. doi: 10.1038/s41598-021-82990-y 33637776 PMC7910492

[38] Liu J Zhang J. Elevated EXO1 expression is associated with breast carcinogenesis and poor prognosis. Ann Transl Med. (2021) 9:135. doi: 10.21037/atm-20-7922 33569437 PMC7867906

[39] He D Li T Sheng M Yang B. Exonuclease 1 (Exo1) participates in mammalian non-homologous end joining and contrib-utes to drug resistance in ovarian cancer. Med Sci Monit. (2020) 26:e918751. doi: 10.12659/MSM.918751 32167078 PMC7092659

[40] Zhou CS Feng MT Chen X Gao Y Chen L Li LD. Exonuclease 1 (EXO1) is a potential prognostic bi-omarker and correlates with immune infiltrates in lung adenocarcinoma. Onco Targets Ther. (2021) 14:1033–48. doi: 10.2147/OTT.S286274 PMC7894803 33623391

[41] Yang G Dong K Zhang Z Zhang E Liang B Chen X. EXO1 plays a carcinogenic role in hepatocellular carci-noma and is related to the regulation of FOXP3. J Cancer. (2020) 11:4917–32. doi: 10.7150/jca.40673 PMC7330697 32626539

[42] Luo F Wang Y Lin D Li J Yang K. Exonuclease 1 expression is associated with clinical progression, metastasis, and sur-vival prognosis of prostate cancer. J Cell Biochem. (2019) 120:11383–9. doi: 10.1002/jcb.v120.7 30775798

[43] Teng PC Huang SP Liu CH Lin TY Cho YC Lai YL. Identification of DNA damage repair-associated prognostic biomarkers for prostate cancer using transcriptomic data analysis. Int J Mol Sci. (2021) 22:11771. doi: 10.3390/ijms222111771 34769200 PMC8584064

[44] Fan J Zhao Y Yuan H Yang J Li T He Z. Phospholipase C-ϵ regulates bladder cancer cells via ATM/EXO1. Am J Cancer Res. (2020) 10:2319–36. PMC7471350 32905533

[45] Song F Qureshi AA Zhan J Amos CI Lee JE Wei Q. Exonuclease 1 (EXO1) gene variation and melanoma risk. DNA Repair. (2012) 11:304–9. doi: 10.1016/j.dnarep.2011.12.005 PMC3274568 22230721

[46] Chen X Kim IK Honaker Y Paudyal SC Koh WK Sparks M. 14-3–3 proteins restrain the Exo1 nuclease to prevent overresection. J Biol Chem. (2015) 290:12300–12. doi: 10.1074/jbc.M115.644005 PMC4424361 25833945

[47] Chen X Paudyal SC Chin RI You Z. PCNA promotes processive DNA end resection by Exo1. Nucleic Acids Res. (2013) 41:9325–38. doi: 10.1093/nar/gkt672 PMC3814391 23939618

[48] Imtiaz H Afroz S Hossain MA Bellah SF Rahman MM Kadir MS. Genetic polymor-phisms in CDH1 and Exo1 genes elevate the prostate cancer risk in Bangladeshi population. Tumour Biol. (2019) 41:1010428319830837. doi: 10.1177/1010428319830837 30880589

[49] Castillejo A Rothman N Murta-Nascimento C Malats N García-Closas M Gómez-Martínez A. TGFB1 and TGFBR1 polymorphic variants in relationship to bladder cancer risk and prognosis. Int J Cancer. (2009) 124:608–13. doi: 10.1002/ijc.v124:3 PMC6896897 19004027

[50] Qi F Du X Zhao Z Zhang D Huang M Bai Y. Tumor mutation burden-associated LINC00638/miR-4732-3p/ULBP1 axis promotes immune escape via PD-L1 in hepatocellular carcinoma. Front Oncol. (2021) 11:729340. doi: 10.3389/fonc.2021.729340 34568062 PMC8456090

[51] Zhu YD Lu MY. Increased expression of TNFRSF14 indicates good prognosis and inhibits bladder cancer proliferation by promoting apoptosis. Mol Med Rep. (2018) 18:3403–10. doi: 10.3892/mmr.2018.9306 30066919

[52] Chen Q Jun H Yang C Yang F Xu Y. The pyroptosis-related risk genes APOBEC3D, TNFRSF14, and RAC2 were used to evaluate prognosis and as tumor suppressor genes in breast cancer. J Oncol. (2022) 2022:3625790. doi: 10.1155/2022/3625790 36059808 PMC9436599

[53] Hughes CE Nibbs RJB. A guide to chemokines and their receptors. FEBS J. (2018) 285:2944–71. doi: 10.1111/febs.2018.285.issue-16 PMC6120486 29637711

[54] Jiang X Liu J Li S Jia B Huang Z Shen J. CCL18-induced LINC00319 promotes proliferation and metastasis in oral squamous cell carcinoma via the miR-199a-5p/FZD4 axis. Cell Death Dis. (2020) 11:777. doi: 10.1038/s41419-020-02978-w 32948745 PMC7501282

[55] Su S Liao J Liu J Huang D He C Chen F. Blocking the recruitment of naive CD4+ T cells reverses immunosuppression in breast cancer. Cell Res. (2017) 27:461–82. doi: 10.1038/cr.2017.34 PMC5385617 28290464

[56] Zhu M Xu W Wei C Huang J Xu J Zhang Y. CCL14 serves as a novel prognostic factor and tumor suppressor of HCC by modulating cell cycle and promoting apoptosis. Cell Death Dis. (2019) 10:796. doi: 10.1038/s41419-019-1966-6 31641099 PMC6805940

[57] Das S Sarrou E Podgrabinska S Cassella M Mungamuri SK Feirt N. Tumor cell entry into the lymph node is controlled by CCL1 chemokine expressed by lymph node lymphatic sinuses. J Exp Med. (2013) 210:1509–28. doi: 10.1084/jem.20111627 PMC3727324 23878309

[58] Zhao X Liu J Ge S Chen C Li S Wu X. Saikosaponin A inhibits breast cancer by regulating th1/th2 balance. Front Pharmacol. (2019) 10:624. doi: 10.3389/fphar.2019.00624 31214035 PMC6558179

[59] De Monte L Reni M Tassi E Clavenna D Papa I Recalde H. Intratumor T helper type 2 cell infiltrate correlates with cancer-associated fibroblast thymic stromal lymphopoietin production and reduced survival in pancreatic cancer. J Exp Med. (2011) 208:469–78. doi: 10.1084/jem.20101876 PMC3058573 21339327

[60] Gan Y Li X Han S Liang Q Ma X Rong P. The cGAS/STING Pathway: A novel target for cancer therapy. Front Immunol. (2022) 12:795401. doi: 10.3389/fimmu.2021.795401 35046953 PMC8761794

