# Supplementary figures and images for: Deciphering the multifaceted role of EXO1 in female-related cancers: implications for prognosis and therapeutic responsiveness
Source: Front Immunol. 2025 May 12;16:1591505. doi: 10.3389/fimmu.2025.1591505 (PMC12107352; doi:10.3389/fimmu.2025.1591505)

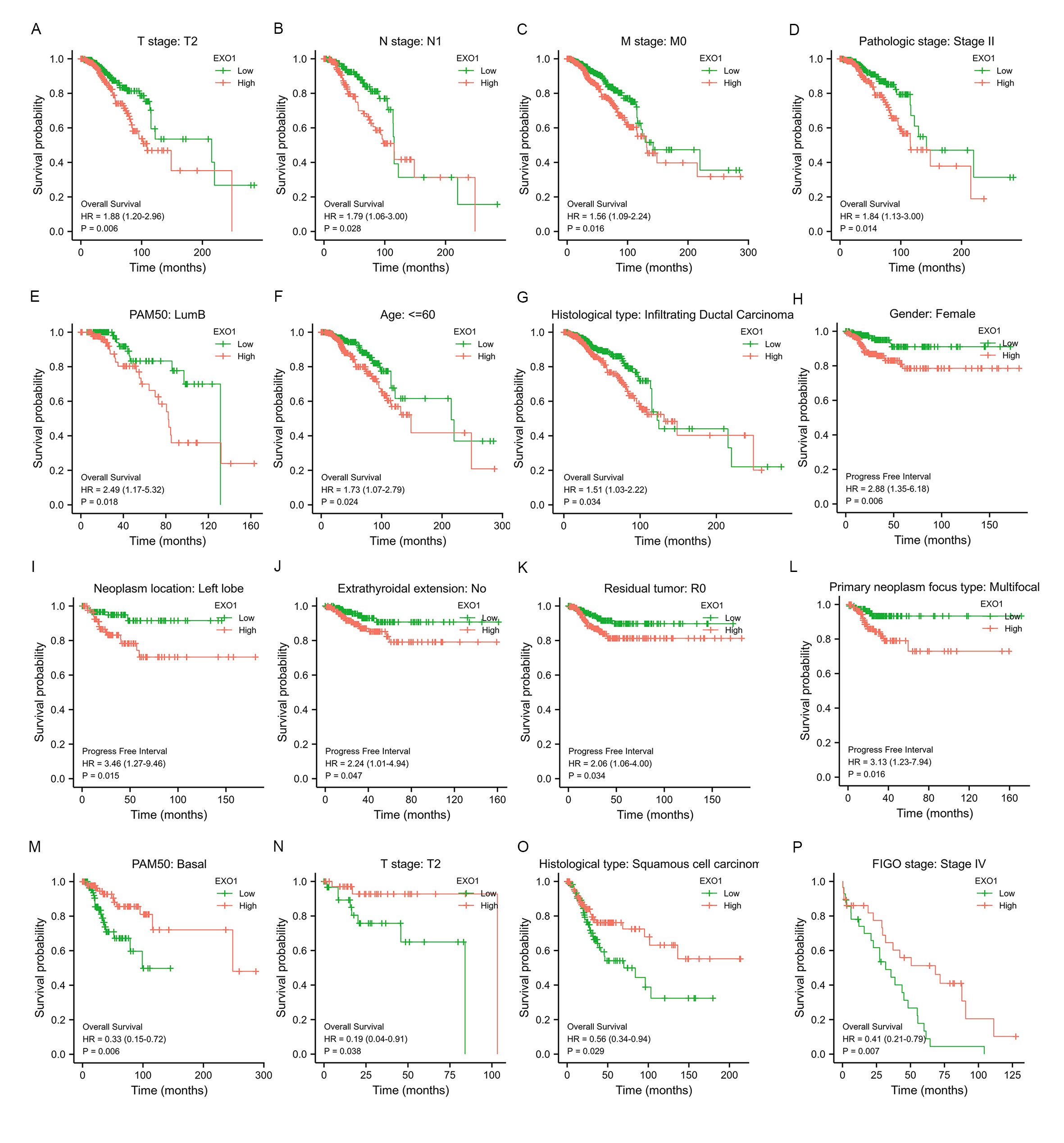

Supplement: Supplementary file 1 [file Image1.tif]

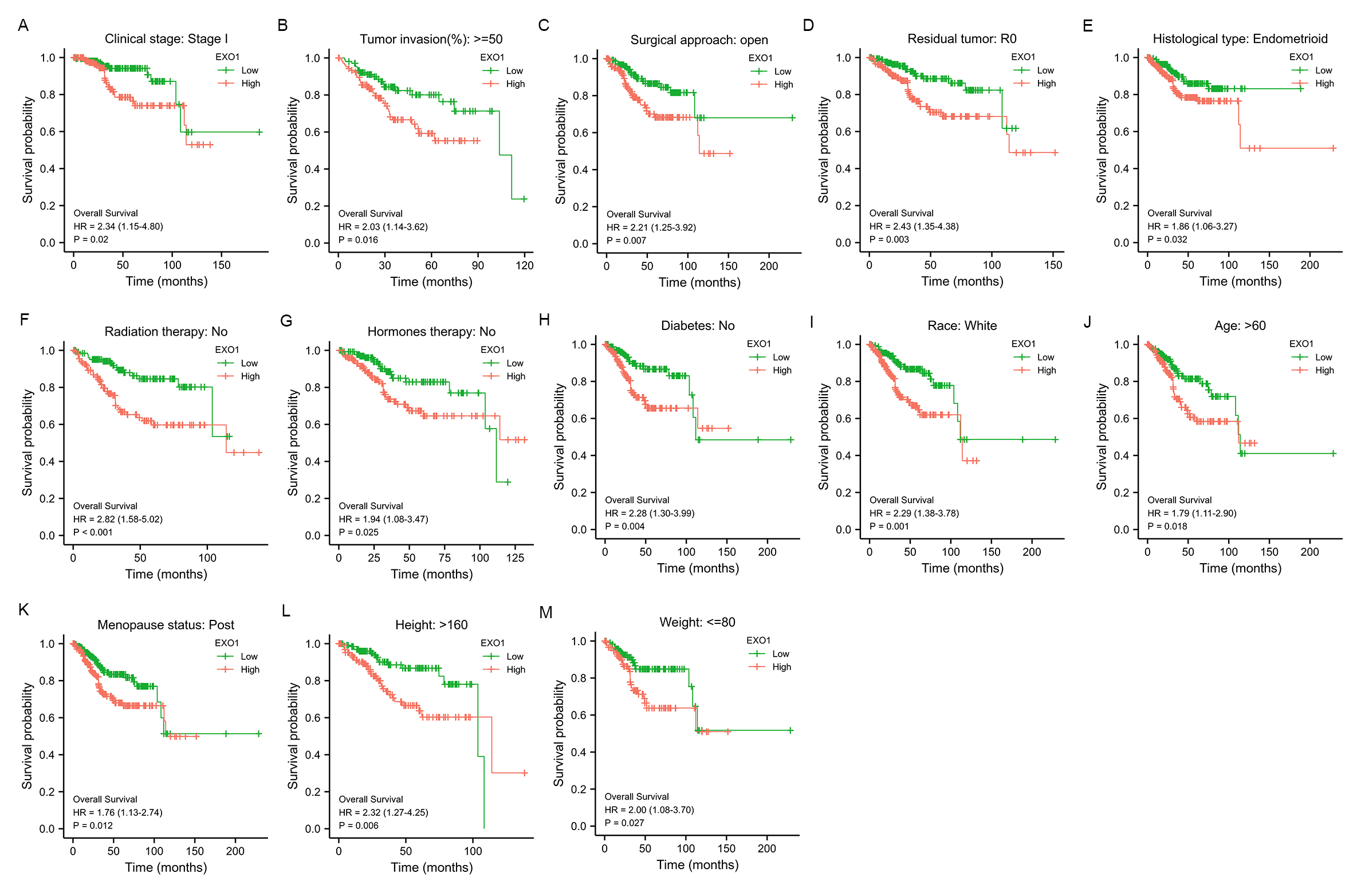

Supplement: Supplementary file 2 [file Image2.tif]
