# Supplementary material for: Deciphering the multifaceted role of EXO1 in female-related cancers: implications for prognosis and therapeutic responsiveness
Source: Front Immunol. 2025 May 12;16:1591505. doi: 10.3389/fimmu.2025.1591505 (PMC12107352; doi:10.3389/fimmu.2025.1591505)
Supplement: Supplementary file 3 [file Table1.docx]

Supplementary Figure 1. Prognostic prediction values of EXO1 in clinicopathological subgroups of female-related cancers. The Kaplan‒Meier plotter was used to examine the association between EXO1 expression and clinical subgroups prognosis in BRCA (A-H), THCA(I-M), CESC (N-O) and OV (P).

Supplementary Figure 2. Prognostic prediction values of EXO1 in clinicopathological subgroups of UCEC. The Kaplan‒Meier plotter was used to examine the association between EXO1 expression and clinical subgroups prognosis.

**Supplementary Table 1** Associations of progression-free interval (PFI) with clinicopathologic characteristics in TCGA patients by univariate and multivariate analyses.

| **Characteristics** | **Univariate analysis** | | **Multivariate analysis** | |
| --- | --- | --- | --- | --- |
|  | **HR (95% CI)** | ***P*-value** | **HR (95% CI)** | ***P-*value** |
| **BRCA** |  |  |  |  |
| T stage |  |  |  |  |
| T1 vs. T2 | 1.613 (1.041-2.499) | 0.032 | 1.280 (0.565-2.900) | 0.554 |
| T1 vs. T3&T4 | 2.851 (1.746-4.654) | <0.001 | 1.301 (0.489-3.459) | 0.598 |
| N stage |  |  |  |  |
| N0 vs. N1 | 1.981 (1.331-2.948) | <0.001 | 1.266 (0.737-2.175) | 0.393 |
| N0 vs. N2 | 2.481 (1.441-4.272) | 0.001 | 1.471 (0.547-3.957) | 0.444 |
| N0 vs. N3 | 4.961 (2.833-8.688) | <0.001 | 2.570 (0.993-6.651) | 0.052 |
| M stage (M0 vs. M1) | 8.315 (4.829-14.315) | <0.001 | 6.545 (1.444-29.672) | 0.015 |
| Pathologic stage |  |  |  |  |
| Stage I vs. Stage III | 3.428 (1.908-6.161) | <0.001 | 1.735 (0.433-6.960) | 0.437 |
| Stage I vs. Stage IV | 14.509 (6.877-30.611) | <0.001 |  |  |
| PR status (Negative vs. Positive) | 0.558 (0.400-0.779) | <0.001 | 0.508 (0.280-0.921) | 0.026 |
| ER status (Negative vs. Positive) | 0.622 (0.436-0.887) | 0.009 | 0.596 (0.307-1.156) | 0.126 |
| EXO1 (Low vs. High) | 1.452 (1.047-2.014) | 0.025 | 1.384 (0.832-2.302) | 0.211 |
| **UCEC** |  |  |  |  |
| Clinical stage |  |  |  |  |
| Stage I vs. Stage III | 2.581 (1.740-3.827) | <0.001 | 2.052 (1.008-4.180) | 0.048 |
| Stage I vs. Stage IV | 6.832 (4.081-11.437) | <0.001 | 3.702 (1.168-11.736) | 0.026 |
| Primary therapy outcome |  |  |  |  |
| PD vs. SD | 0.191 (0.057-0.647) | 0.008 | 0.136 (0.013-1.407) | 0.094 |
| PD vs. PR | 0.291 (0.122-0.692) | 0.005 | 1.002 (0.226-4.436) | 0.998 |
| PD vs. CR | 0.059 (0.036-0.099) | <0.001 | 0.125 (0.035-0.447) | 0.001 |
| Race (Asian vs. Black or African American) | 4.631 (1.106-19.394) | 0.036 | 0.437 (0.082-2.330) | 0.332 |
| Histological type |  |  |  |  |
| Endometrioid vs. Mixed | 2.035 (0.981-4.221) | 0.056 | 12.524 (3.596-43.623) | <0.001 |
| Endometrioid vs. Serous | 2.123 (1.464-3.078) | <0.001 | 3.439 (1.472-8.033) | 0.004 |
| Histologic grade |  |  |  |  |
| G1 vs. G2 | 2.156 (1.015-4.580) | 0.046 | 1.702 (0.579-5.001) | 0.333 |
| G1 vs. G3 | 3.281 (1.708-6.300) | <0.001 | 0.617 (0.193-1.972) | 0.415 |
| Tumor invasion(%) | 1.013 (1.008-1.018) | <0.001 | 1.006 (0.999-1.013) | 0.097 |
| Residual tumor |  |  |  |  |
| R0 vs. R1 | 1.396 (0.607-3.213) | 0.433 | 4.656 (1.603-13.525) | 0.005 |
| R0 vs. R2 | 5.209 (2.821-9.621) | <0.001 | 1.604 (0.386-6.675) | 0.516 |
| Surgical approach (Minimally Invasive vs. open) | 0.629 (0.440-0.899) | 0.011 | 0.773 (0.409-1.460) | 0.427 |
| EXO1 (Low vs. High) | 1.827 (1.280-2.607) | <0.001 | 1.985 (1.082-3.642) | 0.027 |
| **THCA** |  |  |  |  |
| T stage |  |  |  |  |
| T1 vs. T2 | 3.622 (1.219-10.768) | 0.021 | 2.259 (0.541-9.422) | 0.264 |
| T1 vs. T3&T4 | 5.921 (2.097-16.719) | <0.001 | 2.231 (0.431-11.558) | 0.339 |
| M stage (M0 vs. M1) | 7.305 (2.780-19.197) | <0.001 | 4.109 (0.795-21.235) | 0.092 |
| Pathologic stage |  |  |  |  |
| Stage I vs. Stage III | 2.181 (1.130-4.210) | 0.020 | 2.885 (0.569-14.621) | 0.201 |
| Stage I vs. Stage IV | 4.021 (1.996-8.102) | <0.001 | 2.918 (0.373-22.804) | 0.307 |
| Extrathyroidal extension (No vs. Yes) | 1.874 (1.092-3.216) | 0.023 | 1.031 (0.324-3.280) | 0.959 |
| EXO1 (Low vs. High) | 4.045 (2.056-7.958) | <0.001 | 3.120 (1.057-9.209) | 0.039 |
| **OV** |  |  |  |  |
| FIGO stage (Stage I&Stage II vs.Stage IV) | 2.069 (1.135-3.773) | 0.018 | 1.284 (0.546-3.023) | 0.567 |
| Primary therapy outcome (PD vs. CR) | 0.308 (0.198-0.477) | <0.001 | 0.605 (0.374-0.979) | 0.041 |
| Tumor residual (NRD vs. RD) | 1.695 (1.219-2.358) | 0.002 | 0.830 (0.552-1.247) | 0.370 |
| Tumor status (Tumor free vs. With tumor) | 10.045 (5.758-17.526) | <0.001 | 12.072 (5.932-24.566) | <0.001 |
| **CESC** |  |  |  |  |
| T stage (T1 vs. T3&T4) | 3.259 (1.731-6.135) | <0.001 | 4.058 (0.728-22.624) | 0.110 |
| N stage (N0 vs. N1) | 2.108 (1.062-4.187) | 0.033 | 2.238 (0.647-7.735) | 0.203 |
| M stage (M0 vs. M1) | 3.072 (1.154-8.177) | 0.025 | <0.001 (<0.001-Inf) | 0.998 |
| Clinical stage (Stage I vs. Stage IV) | 3.923 (2.111-7.290) | <0.001 | 0.495 (0.031-7.917) | 0.619 |
| Primary therapy outcome |  |  |  |  |
| PD vs. SD | 0.085 (0.011-0.631) | 0.016 | <0.001 (<0.001-Inf) | 0.999 |
| PD vs. PR | 0.270 (0.101-0.722) | 0.009 | 1.000 (1.000-1.000) |  |
| PD vs. CR | 0.064 (0.036-0.115) | <0.001 | 0.031 (0.003-0.368) | 0.006 |
| BMI (<=25 vs. >25) | 0.583 (0.345-0.984) | 0.043 | 0.469 (0.143-1.542) | 0.212 |
| **UCS** |  |  |  |  |
| Clinical stage |  |  |  |  |
| Stage I vs. Stage II&Stage III | 2.320 (1.038-5.183) | 0.040 | 0.285 (0.005-16.580) | 0.545 |
| Stage I vs. Stage IV | 3.036 (1.182-7.800) | 0.021 | 49.606 (0.400-6154.907) | 0.112 |
| Primary therapy outcome (PD vs. SD&PR&CR) | 0.091 (0.036-0.229) | <0.001 | <0.001 (<0.001-0.072) | 0.004 |
| TP53 | 1.375 (0.951-1.987) | 0.090 | 3.726 (1.227-11.319) | 0.020 |
| Residual tumor (R0 vs. R1&R2) | 2.513 (1.071-5.900) | 0.034 | 0.235 (0.009-6.400) | 0.391 |
| Radiation therapy (No vs. Yes) | 0.463 (0.233-0.920) | 0.028 | 80.3 (1.150-5619.6) | 0.043 |
| Peritoneal wash (Negative vs. Positive) | 3.175 (1.306-7.721) | 0.011 | 1.980 (0.129-30.483) | 0.624 |
| Hypertension (No vs. Yes) | 2.247 (1.075-4.694) | 0.031 | 4.598 (0.591-35.778) | 0.145 |

**Supplementary Table 2.** Associations of disease-specific survival (DSS) with clinicopathologic characteristics in TCGA patients by univariate and multivariate analyses.

| **Characteristics** | **Univariate analysis** | | **Multivariate analysis** | |
| --- | --- | --- | --- | --- |
|  | **HR (95% CI)** | ***P*-value** | **HR (95% CI)** | ***P-*value** |
| **BRCA** |  |  |  |  |
| T stage (T1 vs. T3&T4) | 2.581 (1.358-4.902) | 0.004 | 0.419 (0.115-1.528) | 0.187 |
| N stage |  |  |  |  |
| N0 vs. N1 | 3.390 (1.926-5.967) | <0.001 | 1.821 (0.764-4.337) | 0.176 |
| N0 vs. N2 | 3.752 (1.756-8.020) | <0.001 | 3.118 (0.710-13.704) | 0.132 |
| N0 vs. N3 | 7.123 (3.317-15.296) | <0.001 | 5.320 (1.339-21.139) | 0.018 |
| M stage ( M0 vs. M1) | 7.454 (3.988-13.931) | <0.001 | 11.664 (1.493-91.148) | 0.019 |
| Pathologic stage |  |  |  |  |
| Stage I vs. Stage III | 5.006 (2.065-12.137) | <0.001 | 1.615 (0.396-6.584) | 0.504 |
| Stage I vs. Stage IV | 26.261 (9.826-70.183) | <0.001 | 2.548 (0.388-16.744) | 0.330 |
| Histological type (Ductal vs. Lobular) | 0.471 (0.226-0.982) | 0.045 | 0.554 (0.195-1.576) | 0.268 |
| PR status (Negative vs. Positive) | 0.519 (0.334-0.807) | 0.004 | 0.584 (0.232-1.472) | 0.254 |
| ER status (Negative vs. Positive) | 0.559 (0.351-0.891) | 0.015 | 0.441 (0.162-1.198) | 0.108 |
| EXO1 (Low vs. High) | 1.698 (1.097-2.628) | 0.017 | 2.099 (1.003-4.391) | 0.049 |
| **UCEC** |  |  |  |  |
| Clinical stage |  |  |  |  |
| Stage I vs. Stage III | 5.935 (3.160-11.145) | <0.001 | 3.451 (1.010-11.796) | 0.048 |
| Stage I vs. Stage IV | 16.815 (8.274-34.173) | <0.001 | 3.494 (0.672-18.153) | 0.137 |
| Primary therapy outcome (PD vs. CR) | 0.062 (0.032-0.121) | <0.001 | 0.138 (0.035-0.543) | 0.005 |
| Histological type |  |  |  |  |
| Endometrioid vs. Mixed | 3.981 (1.651-9.599) | 0.002 | 8.919 (1.962-40.543) | 0.005 |
| Endometrioid vs. Serous | 3.493 (2.071-5.891) | <0.001 | 1.743 (0.678-4.484) | 0.249 |
| Tumor invasion(%) | 1.013 (1.008-1.017) | <0.001 | 1.004 (0.994-1.014) | 0.425 |
| Residual tumor |  |  |  |  |
| R0 vs. R1 | 2.705 (1.049-6.974) | 0.040 | 7.438 (2.196-25.193) | 0.001 |
| R0 vs. R2 | 9.442 (4.728-18.856) | <0.001 | 3.884 (0.955-15.806) | 0.058 |
| Radiation therapy (No vs. Yes) | 0.599 (0.351-1.021) | 0.060 | 0.248 (0.085-0.724) | 0.011 |
| EXO1 (Low vs. High) | 1.796 (1.080-2.988) | 0.024 | 2.155 (0.918-5.060) | 0.078 |
| **OV** |  |  |  |  |
| FIGO stage (Stage I&Stage II vs. Stage IV) | 2.832 (1.114-7.198) | 0.029 | 2.392 (0.556-10.286) | 0.241 |
| Primary therapy outcome |  |  |  |  |
| PD vs. SD | 0.443 (0.217-0.906) | 0.026 | 0.561 (0.260-1.210) | 0.141 |
| PD vs. CR | 0.145 (0.088-0.240) | <0.001 | 0.181 (0.104-0.316) | <0.001 |
| Race (Asian&Black or African American vs. White) | 0.592 (0.370-0.946) | 0.028 | 1.004 (0.552-1.827) | 0.989 |
| ERBB2 (Low vs. High) | 1.435 (1.085-1.898) | 0.011 | 1.265 (0.906-1.766) | 0.167 |
| Tumor residual (NRD vs. RD) | 2.572 (1.580-4.187) | <0.001 | 1.925 (1.111-3.333) | 0.019 |
| **CESC** |  |  |  |  |
| T stage (T1 vs. T3&T4) | 5.118 (2.462-10.637) | <0.001 | 4.541 (0.526-39.197) | 0.169 |
| N stage (N0 vs. N1) | 3.544 (1.572-7.987) | 0.002 | 1.860 (0.500-6.911) | 0.354 |
| M stage (M0 vs. M1) | 4.290 (1.401-13.137) | 0.011 | <0.001 (<0.001-Inf) | 0.999 |
| Primary therapy outcome (PD vs. CR) | 0.034 (0.017-0.069) | <0.001 | 0.372 (0.045-3.066) | 0.358 |

**Supplementary Table 3.** Differentially expressed genes (DEGs) between high and low EXO1 expression groups.

| **Cancer Type** | **Total DEGs** | **Upregulated** | **Downregulated** |
| --- | --- | --- | --- |
| **BRCA** | 629 | 274 | 355 |
| **UCEC** | 120 | 37 | 83 |
| **THCA** | 65 | 54 | 12 |
| **OV** | 78 | 34 | 44 |
| **CESC** | 182 | 27 | 155 |
| **UCS** | 431 | 178 | 253 |

**Supplementary Table 4.** Enriched GO terms and KEGG classification of DEGs.

| **ONTOLOGY** | **ID** | **Description** | **p.adj** | **Count** | **zscore** |
| --- | --- | --- | --- | --- | --- |
| **BRCA** |  |  |  |  |  |
| BP | GO:0019730 | antimicrobial humoral response | 0.005 | 9 | 1.667 |
| BP | GO:0051930 | regulation of sensory perception of pain | 0.026 | 5 | 1.342 |
| BP | GO:0001580 | detection of chemical stimulus involved in sensory perception of bitter taste | 0.026 | 5 | 0.447 |
| BP | GO:0050913 | sensory perception of bitter taste | 0.031 | 5 | 0.447 |
| BP | GO:0031424 | keratinization | 0.031 | 10 | 0.632 |
| CC | GO:0045095 | keratin filament | 0.014 | 7 | 0.378 |
| CC | GO:0045111 | intermediate filament cytoskeleton | 0.031 | 10 | 1.265 |
| CC | GO:0005882 | intermediate filament | 0.031 | 9 | 1.000 |
| KEGG | hsa04080 | Neuroactive ligand-receptor interaction | 0.002 | 12 | 0.577 |
| **UCEC** |  |  |  |  |  |
| BP | GO:0019731 | antibacterial humoral response | 0.000 | 5 | -2.236 |
| BP | GO:0002385 | mucosal immune response | 0.003 | 4 | -2.000 |
| BP | GO:0002251 | organ or tissue specific immune response | 0.003 | 4 | -2.000 |
| BP | GO:0006959 | humoral immune response | 0.006 | 8 | -2.828 |
| BP | GO:0045861 | negative regulation of proteolysis | 0.027 | 7 | -2.646 |
| BP | GO:0048871 | multicellular organismal homeostasis | 0.027 | 8 | -2.121 |
| MF | GO:0004866 | endopeptidase inhibitor activity | 0.000 | 7 | -2.646 |
| MF | GO:0061134 | peptidase regulator activity | 0.000 | 7 | -2.646 |
| MF | GO:0005179 | hormone activity | 0.002 | 5 | 1.342 |
| MF | GO:0004857 | enzyme inhibitor activity | 0.008 | 7 | -2.646 |
| KEGG | hsa04080 | Neuroactive ligand-receptor interaction | 0.002 | 7 | 1.134 |
| KEGG | hsa04970 | Salivary secretion | 0.004 | 4 | -2.000 |
| KEGG | hsa04270 | Vascular smooth muscle contraction | 0.012 | 4 | 0.000 |
| **THCA** |  |  |  |  |  |
| BP | GO:0006959 | humoral immune response | 0.000 | 14 | 3.207 |
| BP | GO:0042113 | B cell activation | 0.000 | 12 | 3.464 |
| BP | GO:0016064 | immunoglobulin mediated immune response | 0.000 | 10 | 3.162 |
| BP | GO:0019724 | B cell mediated immunity | 0.000 | 10 | 3.162 |
| BP | GO:0002449 | lymphocyte mediated immunity | 0.000 | 11 | 3.317 |
| BP | GO:0002920 | regulation of humoral immune response | 0.000 | 8 | 2.828 |
| BP | GO:0002673 | regulation of acute inflammatory response | 0.000 | 7 | 2.646 |
| BP | GO:0002697 | regulation of immune effector process | 0.000 | 10 | 3.162 |
| BP | GO:0050900 | leukocyte migration | 0.008 | 7 | 2.646 |
| CC | GO:0019814 | immunoglobulin complex | 0.000 | 7 | 2.646 |
| CC | GO:0009897 | external side of plasma membrane | 0.000 | 9 | 2.333 |
| CC | GO:0030016 | myofibril | 0.003 | 5 | 2.236 |
| CC | GO:0045111 | intermediate filament cytoskeleton | 0.024 | 4 | 1.000 |
| MF | GO:0003823 | antigen binding | 0.001 | 6 | 2.449 |
| MF | GO:0034987 | immunoglobulin receptor binding | 0.004 | 4 | 2.000 |
| KEGG | hsa04640 | Hematopoietic cell lineage | 0.002 | 4 | 2.000 |
| **OV** |  |  |  |  |  |
| BP | GO:0031016 | pancreas development | 0.002 | 4 | 2.000 |
| BP | GO:0000395 | mRNA 5'-splice site recognition | 0.002 | 3 | -1.732 |
| BP | GO:0045292 | mRNA cis splicing, via spliceosome | 0.009 | 3 | -1.732 |
| BP | GO:0048732 | gland development | 0.047 | 5 | 2.236 |
| CC | GO:0005685 | U1 snRNP | 0.001 | 3 | -1.732 |
| CC | GO:0097525 | spliceosomal snRNP complex | 0.009 | 3 | -1.732 |
| CC | GO:0030532 | small nuclear ribonucleoprotein complex | 0.009 | 3 | -1.732 |
| CC | GO:0120114 | Sm-like protein family complex | 0.009 | 3 | -1.732 |
| MF | GO:0036002 | pre-mRNA binding | 0.000 | 4 | -1.000 |
| MF | GO:0030627 | pre-mRNA 5'-splice site binding | 0.000 | 3 | -1.732 |
| MF | GO:0005179 | hormone activity | 0.012 | 3 | 1.732 |
| MF | GO:0005184 | neuropeptide hormone activity | 0.012 | 2 | 1.414 |
| KEGG | hsa03040 | Spliceosome | 0.008 | 3 | -1.732 |
| KEGG | hsa03013 | RNA transport | 0.008 | 3 | -1.732 |
| **CESC** |  |  |  |  |  |
| BP | GO:0009913 | epidermal cell differentiation | 0.011 | 10 | -3.162 |
| BP | GO:0008544 | epidermis development | 0.015 | 11 | -3.317 |
| BP | GO:0001894 | tissue homeostasis | 0.039 | 7 | -2.646 |
| CC | GO:0045177 | apical part of cell | 0.000 | 15 | -3.357 |
| CC | GO:0016324 | apical plasma membrane | 0.000 | 12 | -2.887 |
| CC | GO:0098858 | actin-based cell projection | 0.048 | 6 | -2.449 |
| MF | GO:0004866 | endopeptidase inhibitor activity | 0.013 | 7 | -2.646 |
| MF | GO:0061134 | peptidase regulator activity | 0.019 | 7 | -2.646 |
| MF | GO:0030246 | carbohydrate binding | 0.038 | 7 | -2.646 |
| KEGG | hsa04950 | Maturity onset diabetes of the young | 0.026 | 3 | -1.732 |
| KEGG | hsa00053 | Ascorbate and aldarate metabolism | 0.027 | 3 | 0.577 |
| **UCS** |  |  |  |  |  |
| BP | GO:0006959 | humoral immune response | 0.000 | 54 | -7.348 |
| BP | GO:0006956 | complement activation | 0.000 | 42 | -6.481 |
| BP | GO:0016064 | immunoglobulin mediated immune response | 0.000 | 42 | -6.481 |
| BP | GO:0019724 | B cell mediated immunity | 0.000 | 42 | -6.481 |
| BP | GO:0002526 | acute inflammatory response | 0.000 | 39 | -6.245 |
| BP | GO:0006909 | phagocytosis | 0.000 | 44 | -6.030 |
| CC | GO:0019814 | immunoglobulin complex | 0.000 | 47 | -6.856 |
| CC | GO:0009897 | external side of plasma membrane | 0.000 | 30 | -5.477 |
| CC | GO:0071682 | endocytic vesicle lumen | 0.003 | 4 | -2.000 |
| CC | GO:0042101 | T cell receptor complex | 0.006 | 8 | -2.828 |
| CC | GO:0016459 | myosin complex | 0.020 | 5 | 2.236 |
| MF | GO:0034987 | immunoglobulin receptor binding | 0.000 | 26 | -5.099 |
| MF | GO:0042379 | chemokine receptor binding | 0.002 | 7 | -2.646 |
| MF | GO:0048020 | CCR chemokine receptor binding | 0.012 | 5 | -2.236 |
| MF | GO:0008009 | chemokine activity | 0.017 | 5 | -2.236 |
| MF | GO:0042056 | chemoattractant activity | 0.033 | 4 | -1.000 |
| KEGG | hsa04657 | IL-17 signaling pathway | 0.000 | 11 | -3.317 |
| KEGG | hsa04060 | Cytokine-cytokine receptor interaction | 0.008 | 11 | -2.714 |

**Supplementary Table 5.** Spearman correlation analysis between EXO1 expression level and infiltration levels of 24 indicated immune cells in tumor tissues.

| **Cancer Type** | **Positive Correlation** | **Negative Correlation** |
| --- | --- | --- |
| **BRCA** | aDC, B cells, Macrophages, NK CD56dim, T helper, Tgd, Th1, Th2, TReg | CD8 T cells, Cytotoxic cells, Eosinophils, iDC, Mast cells, NK CD56bright, NK cells, pDC, Th17 |
| **UCEC** | T helper, Tcm, Tgd, Th2 | B cells, CD8 T cells, Cytotoxic cells, DC, iDC, Mast cells, Neutrophils, NK CD56bright, NK CD56dim, pDC, T cells, TFH, Th17 |
| **THCA** | aDC, B cells, CD8 T cells, DC, iDC, Macrophages, Mast cells, Neutrophils, NK CD56dim, T helper, Tcm, Tem, TFH, Tgd, Th1, Th2, TReg | NK CD56bright, NK cells, pDC, Th17 |
| **OV** | T helper, Th2 | CD8 T cells, Cytotoxic cells, DC, iDC, Mast cells, NK CD56bright, pDC, T cells, Th1, Th17 |
| **CESC** | T helper, Tcm, Th2 | Cytotoxic cells, DC, iDC, Mast cells, Neutrophils, pDC, TFH, Th1 |
| **UCS** | Tgd, Th2 | aDC, B cells, CD8 T cells, Cytotoxic cells, DC, iDC, Macrophages, Mast cells, Neutrophils, NK CD56dim, pDC, T cells, Th17 |

**Supplementary Table 6.** Spearman correlation analysis between EXO1 expression level and immune inhibitors and immune stimulators in tumor tissues.

| **Cancer Type** | **Immune Inhibitors** | | **Immune Stimulators** | |
| --- | --- | --- | --- | --- |
|  | **Positive** | **Negative** | **Positive** | **Negative** |
| **BRCA** | BTLA, CD160, CD274, CTLA4, HAVCR2, IDO1, IL10, IL10RB, LAG3, PDCD1LG2, TGFBR1, TIGIT | CSF1R, KDR, KIR2DL1, KIR2DL3, LGALS9, PDCD1, TGFB1, VTCN1 | CD276, CD80, CXCR4, ENTPD1, ICOS, IL2RA, IL6R, MICB, PVR, TNFRSF13C, TNFRSF9, TNFSF13B, TNFSF15, TNFSF4, ULBP1 | CD70, CXCL12, KLRK1, LTA, NT5E, TMIGD2, TNFRSF14, TNFRSF18, TNFRSF25, TNFRSF4, TNFSF9 |
| **UCEC** | ADORA2A, CD160, CD274, IL10, IL10RB, PDCD1LG2, TGFBR1, TIGIT | CSF1R, KDR, LGALS9, PDCD1, TGFB1, TNFRSF14, TNFRSF4 | CD276, CD80, CXCR4, ENTPD1, ICOSLG, IL2RA, IL6R, MICB, PVR, TNFRSF13C, TNFSF18, TNFSF4, ULBP1 | CD40LG, CD70, CXCL12, HHLA2, ICOS, KLRC1, LTA, TMIGD2, TNFRSF14, TNFRSF17, TNFRSF25, TNFRSF4 |
| **THCA** | ADORA2A, BTLA, CD160, CD244, CD274, CSF1R, CTLA4, HAVCR2, IDO1, IL10, LAG3, LGALS9, PDCD1LG2, TGFB1, TGFBR1, TIGIT, VTCN1 | CD96, KDR, PDCD1, TNFRSF14 | CD28, CD276, CD80, CXCR4, ENTPD1, ICOS, ICOSLG, IL2RA, IL6R, KLRK1, MICB, PVR, TNFRSF13C, TNFRSF9, TNFSF13B, TNFSF15, TNFSF4, ULBP1 | CD40, CD48, CD70, HHLA2, KLRC1, LTA, NT5E, TNFRSF14, TNFRSF18, TNFRSF25 |
| **OV** | ADORA2A, CD274, IL10, TGFBR1 | CSF1R, HAVCR2, IDO1, KDR, LGALS9, PDCD1, TGFB1 | CD276, CD28, CD80, CXCR4, ENTPD1, IL6R, MICB, PVR, TNFRSF13C, ULBP1 | CD40LG, CD70, TMIGD2, TNFRSF14, TNFSF13B, TNFSF9 |
| **CESC** | ADORA2A, BTLA, CD274, TGFBR1 | CSF1R, KDR, NT5E, TNFRSF14 | MICB, PVR, TNFRSF13C, TNFSF4, ULBP1 | CD40, CD70, KLRC1, TMIGD2, TNFRSF14, TNFSF9 |
| **UCS** | ADORA2A | CSF1R, HAVCR2, IDO1, KIR2DL1, LAG3, LGALS9, PDCD1, TIGIT, TNFRSF14, TNFSF15 | ENTPD1, ULBP1 | CD40LG, CD70, IL2RA, KLRC1, TNFRSF14, TNFRSF4 |

**Supplementary Table 7.** Spearman correlation analysis between EXO1 expression level and chemokines and chemokine receptors in tumor tissues.

| **Cancer Type** | **Chemokines** | | **Chemokine Receptors** | |
| --- | --- | --- | --- | --- |
|  | **Positive** | **Negative** | **Positive** | **Negative** |
| **BRCA** | CCL1, CCL2, CCL3, CCL4, CCL5, CCL7, CCL8, CCL13, CCL18, CCL20, CXCL1, CXCL3, CXCL5, CXCL6, CXCL8, CXCL9, CXCL10, CXCL11, CXCL16, XCL1 | CCL14, CCL16, CCL19, CCL21, CCL28, CXCL14 | CCR1, CCR8, CXCR4, CXCR6 | CCR10, CXCR1, CXCR2, CXCR3, CX3CR1 |
| **UCEC** | CCL7, CCL8, CCL11, CCL13, CCL18, CCL28, CXCL10, CXCL11 | CCL14, CCL16, CCL17, CCL19, CCL20, CCL21, CXCL2, CXCL14, CXCL17, XCL2 | CCR1, CCR4, CCR8, CXCR4 | CCR3, CCR7, CCR9, CXCR1, CXCR2, CXCR3, CX3CR1 |
| **THCA** | CCL1, CCL2, CCL3, CCL4, CCL5, CCL7, CCL8, CCL11, CCL13, CCL18, CCL19, CCL20, CCL21, CCL22, CCL23, CCL24, CXCL1, CXCL2, CXCL3, CXCL5, CXCL6, CXCL8, CXCL9, CXCL10, CXCL11, CXCL13, CXCL16, CXCL17, XCL1, XCL2 | CCL14, CCL16 | CCR1, CCR2, CCR3, CCR4, CCR5, CCR6, CCR7, CCR8, CCR9, CXCR4, CXCR5, CXCR6, XCR1 | CXCR3, CX3CR1 |
| **OV** | CCL7, CCL8, CCL25, CCL28, CXCL5 | CCL14, CCL17, CXCL17, XCL2 | CCR8, CCR10, CXCR4 | CCR3, CX3CR1 |
| **CESC** | CCL28 | CCL14, CCL16, CCL20 | CCR8 | CCR3, CCR10, CXCR1 |
| **UCS** | - | CCL2, CCL3, CCL4, CCL5, CCL7, CCL8, CCL14, CXCL14 | - | CCR1, CCR3, CCR5, CXCR3, CX3CR1 |
